# Comparing measurements of lithium treatment efficacy in people with bipolar disorder: systematic review and meta-analysis

**DOI:** 10.1192/bjo.2023.64

**Published:** 2023-05-25

**Authors:** Andrea Ulrichsen, Elliot Hampsey, Rosie H. Taylor, Romayne Gadelrab, Rebecca Strawbridge, Allan H. Young

**Affiliations:** Institute of Psychiatry, Psychology and Neuroscience, King's College London, UK; Institute of Psychiatry, Psychology and Neuroscience, King's College London, UK; and South London and Maudsley NHS Foundation Trust, UK

**Keywords:** Bipolar affective disorders, lithium, response, systematic review, meta-analysis

## Abstract

**Background:**

Lithium has long been recognised as an effective treatment for bipolar disorder. Its relative efficacy has been measured with a diverse range of clinical outcomes, resulting in differences in efficacy reporting that have not been systematically reviewed.

**Aims:**

We aimed to identify and compare the various measures of lithium efficacy employed in interventional studies for people with bipolar disorder.

**Method:**

Database (PubMed, Web of Science) and hand searches were performed to identify studies that assessed a clinical response in patients with bipolar disorder who received lithium, up to the end of 2021. We included primary human interventional studies without excluding specific study designs, bipolar disorder subtypes, duration or dosage of lithium treatment. Continuous outcome effects were meta-analysed; binary outcomes were synthesised visually and narratively. The Cochrane risk-of-bias tool was used to assess study-level risk of bias.

**Results:**

Seventy-one studies were included (*N* = 30 542). Approximately two-thirds of participants attained a clinically significant improvement in manic or depressive symptoms, and over 50% achieved remission. About a third required hospital admission (study length 2–12 years) and around 50% needed further treatment to stay well or had recurrence of symptoms; the latter two outcomes tended to be assessed over long-term maintenance periods.

**Conclusions:**

An abundance of measurements have been used to assess lithium's clinical effects, across several study designs. Despite the resultant high heterogeneity, an overall picture of lithium's effects emerges that supports previous literature; between half and two-thirds of patients respond well to lithium across varying outcome measures, baseline mood states, study durations and bipolar disorder subtypes.

## Lithium: pros and cons

Lithium has long been acknowledged as the gold standard treatment for bipolar disorder,^1^ not only for its mood-stabilising effect (especially when taken long term), but also as the mood stabiliser with the best evidence for anti-suicidal efficacy.^2–4^ This is pertinent as a recent systematic review found between 4 and 19% of people with bipolar disorder die by suicide, and 20–60% have at least one suicide attempt in their lifetime.^5^ However, the side-effect profile of lithium is often cited as a reason for low prescription rates, including kidney and thyroid toxicity, as well as concerns about hyperparathyroidism and general health side-effects like nausea and weight gain.^6,7^ These reasons could explain why a review from 2007 found that participants receiving lithium treatment in studies were twice as likely to withdraw than participants receiving sodium valproate or lamotrigine.^8^ To mitigate toxicity, lithium treatment requires regular monitoring, which can make this treatment less appealing than other drugs recommended for bipolar disorder. For example, a recent USA survey of clinicians found that side-effects (61.5%), and the time costs of monitoring these (52.3%), were the most frequent obstacles to prescribing lithium.^9^

Lithium is known for its effectiveness at preventing relapse of affective episodes, particularly mania,^10^ with its effect against depressive episodes appearing less robust.^11,12^ A recent systematic review on the efficacy of lithium treatment compared with other treatment options in RCTs found lithium to be effective in both the treatment of acute mania (with and without psychotic symptoms) and preventing manic episodes.^11^ Other reviews have found maintenance lithium treatment to improve clinical outcomes compared with other first-line therapies and placebo.^10,13^

## Assessment of clinical response

The approaches and assessments employed to measure lithium efficacy vary greatly across the literature. Commonly, in short-term studies, effect is measured by severity scales of mania (e.g. the Young Mania Rating Scale; YMRS^14^) or depression (e.g. the Montgomery-Åsberg Depression Rating Scale; MADRS^15^), or by using more holistic judgement of a participant's illness and functioning, such as the Clinical Global Impression Scale (CGI).^16^ In contrast, long-term, observational or register-based studies often employ outcome measures such as number of admissions to hospital post-treatment, or rates of treatment discontinuation. In relation to lithium treatment specifically, the Retrospective Assessment of the Lithium Response Phenotype Scale, or ‘Alda scale’,^17^ is a purpose-designed tool for the retrospective evaluation of lithium treatment efficacy, categorising patients as either good responders, partial responders or non-responders to lithium.^18^ It has been reported that around a third of patients, even over a 10-year period, are excellent lithium responders (no episode relapse).^19,20^ Similarly, a 5-year prospective study (*n* = 402) concluded that around a third had stopped taking lithium, and the remaining patients still taking lithium were split between having or not having further mood episodes (38% and 23%, respectively).^21^ A retrospective study using continuous scoring models to assess degree of improvement found around a third of patients were either non-responders, partial responders or full responders.^22^ It is presently unclear whether efficacy rates are consistent across the diverse outcomes being reported in clinical and academic reports. Using various assessment tools, the definition of response also varies. Nierenberg and DeCecco suggested that there should be greater focus on response or remission criteria (response often defined as 50% or higher change in mood scores^23^) than on individual changes in mood scores,^24^ as the former are more clinically relevant.

## Aims and objectives

The primary aim of the present review was to identify and compare efficacy estimates for the different clinical outcome measures used in interventional and observational studies to examine lithium's efficacy in bipolar disorders. To the best of our knowledge, the differences in measures used and consequent comparisons of outcomes have not yet been subject to review in this way. The secondary aim was to determine if lithium efficacy differs across the literature when isolating specific subgroups (e.g. by bipolar disorder subtype or mood state at baseline). This review builds on the many syntheses previously published on lithium's clinical effects, and attempts to highlight which ways such an effect can be measured, to help nuance reports of treatment effect.

## Method

This study was preregistered with the International Prospective Register of Systematic Reviews (PROSPERO; registration identifier CRD42020177329),^25^ and followed the PRISMA guidelines for systematic reviews.^26^ See Supplementary Data 1(a) and 1(b) available at https://doi.org/10.1192/bjo.2023.64 for the checklist.

### Search strategy

Databases Medline/PubMed and Web of Science were used to identify relevant papers for the review, using the following search terms: (bipolar disorder* or bipolar affective disorder* or manic-depressive disorder*) AND (lithium) NOT (meta-analysis or review) AND (individual or people or patient or subject or participant or human). See Supplementary Data 2 for complete search strings. The search was not limited by publication date and covered up until the search date of 10 November 2021, at which time hand-searching of relevant articles and citation lists was also conducted. Duplicate records were removed manually by comparing title and abstracts. Eligibility was assessed initially through screening of title and abstract by reviewers in the Rayyan open-source systematic review software (Rayyan Systems Inc., Cambridge, MA, USA; https://www.rayyan.ai/),^27^ and potentially eligible papers were thereafter assessed in full (see Supplementary Data 3 for the Population, Intervention, Comparison, Outcomes and Study (PICOS) table used to assess eligibility). Where multiple papers reported on same study, the study with highest number of participants was included. Title/abstract reviews, full-text reviews and later data extraction were completed independently by a minimum of two authors (A.U., E.H., R.H.T.) using the Rayyan software, with any disagreements settled by senior authors R.S. and A.H.Y.

### Study eligibility

Randomised controlled trials (RCTs), non-randomised controlled trials (NRTs), register studies and naturalistic clinical studies were included in the review, and any individual case studies were excluded. Only studies that were available in English were included. Studies were not limited by any geographical region, treatment duration or number of participants.

### Participant criteria

Participants had to be classified as having bipolar disorder, using either a recognised classification system or by clinical assessment. There was no requirement of any specific subtype or mood state, and participants were not excluded for any comorbid disorder. Included studies had to include adult participants, but three studies that included both adults and participants under 18 years of age were also included (McNamara:^28^ age 15–35 years, mean 18.5 ± 4.2; Strakowski:^29^ age 13–35 years, mean 16, s.d. 2; Tandon:^30^ age 16–65, no mean reported).

### Intervention

Any studies that investigated a change in any measurement from baseline to an end-point were included, provided the measure was related to bipolar disorder illness severity. At least a subset of study participants in each study had to be treated with lithium (with data reported on these), but any dose and duration were accepted, with no requirement for lithium monotherapy; although if participants did not receive monotherapy lithium, they had to be stabilised on other concomitant medications before receiving the lithium intervention. The end-point was defined as the end of lithium treatment or the end of the study. If a study reported results on several defined end-points (e.g. after 5 years and 10 years^31^), then results from all time points were included. If participants were switched or had any other medication added to their lithium treatment, the end-point was defined as the time of change in treatment.

### Outcome measure

The primary outcomes of the review were to determine the outcome measures used to assess lithium treatment efficacy and compare the rates of treatment efficacy according to those measures. Secondary outcomes were to compare relevant subsets of data, such as duration of treatment, study design (i.e. RCT/NRT), baseline mood state, specific bipolar diagnosis or the individual scales used to examine efficacy between studies.

### Data collection process

Three independent authors (A.U., E.H., R.H.T.) completed the data extraction: two authors completed data extraction for each paper, with a third author either confirming agreement or resolving any clear errors. Active discrepancies between authors were resolved by senior authors (R.S., A.H.Y.). When studies had several groups of patients or several interventions, we extracted data for only the bipolar group and/or the lithium group where possible. If a study started with lithium treatment alone and later (per study design) augmented lithium with other treatment(s), only the time of lithium treatment alone was analysed. If more than one lithium efficacy measure was included in the study (e.g. YMRS and MADRS), both measures were included in relevant analyses. Given that studies frequently had multiple measurements to test efficacy, one study participant can provide several data points of results (e.g. mania response and depression response).

Risk of bias (RoB) for all included studies regardless of study design were assessed with the Cochrane RoB tool for RCTs, which was a change from the protocol. The initial plan to assess RoB in NRTs by using a different tool reduced the comparability between RoB ratings between NRTs and RCTs, because of the different scoring criteria. Therefore, to maximise RoB comparability between studies, we used the Cochrane RoB tool for RCTs for the NRTs as well (excluding the randomisation items). Initially this reduced the possibility for the NRTs to have a low RoB, given the fewer elements for NRTs to demonstrate low RoB. We accounted for this in our scoring key (see Supplementary Data 4.4(c–e)), where NRTs had a somewhat lower threshold to be classified as low RoB.

### Data analysis

Eligible studies were divided into three groups: RCTs, two-arm NRTs and one-arm NRTs. Study characteristics (e.g. demographics, study design) were summarised by calculating means of both overall studies and of the three groups individually, and by narratively summarising results. The results of lithium efficacy and the measurements of this effect were split in two groups, depending on the type of data: binary results and continuous results. Binary assessments of lithium efficacy (e.g. proportion of responders) were analysed by categorising the results as follows: positive binary response (e.g. 50% improvement on mood scales), remission (low mood scores, e.g. YMRS score <8) or negative binary response (e.g. hospital admission), using positive or negative outcome to reflect measurements of desired or undesired outcomes, respectively. Proportions were then pooled and visually summarised. For each comparison, pooled response/remission rates are calculated regardless of study type, and are based on the primary end-point.

Outcome measures were only included in meta-analysis when employed in three or more studies; otherwise, these were summarised narratively in text and tables. Studies that reported continuous data for lithium efficacy with respect to symptoms of depression, mania or global impression were included in a within-participant meta-analysis. Change between pre-lithium and end-point (e.g. pre- and post-treatment mean, change in score, plus variability) were inputted into Comprehensive Meta Analysis software, version 3 for Windows (Biostat Inc., Englewood, NJ, USA; https://www.meta-analysis.com/).^32^ Within-participant, random-effects meta-analyses were then conducted with Comprehensive Meta Analysis software, providing pooled standardised mean difference effect sizes (Hedges’ *g*-statistic) with 95% confidence intervals and *I*^2^-statistic to denote heterogeneity between included studies. As secondary comparisons, we explored subgroups to explore heterogeneity. This included RCTs versus NRTs, baseline mood state, duration of treatment and bipolar disorder type. For primary analyses, the efficacy outcomes of depression, mania or global impression were grouped together regardless of specific measurement scale (e.g. MADRS, Hamilton Rating Scale for Depression; HRSD); these were explored individually in secondary analyses.

## Results

### Study selection

**Fig. 1:**
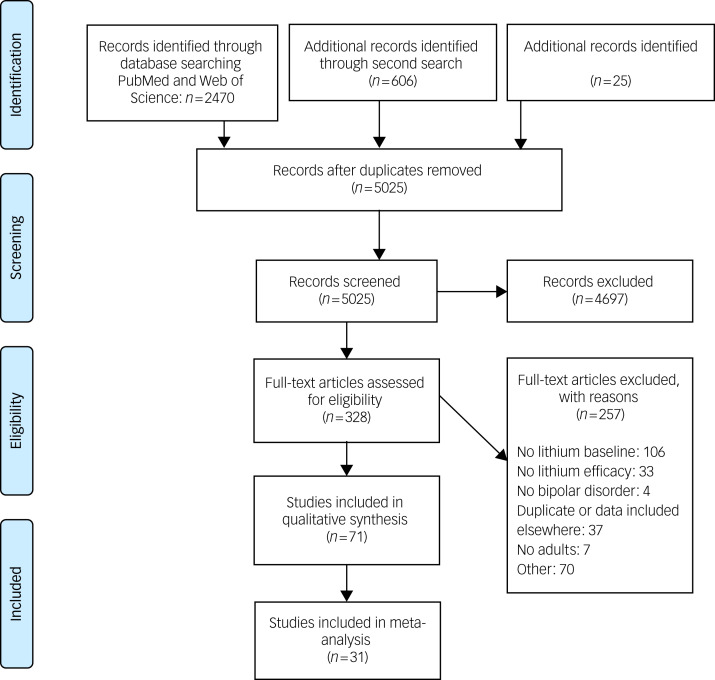
Preferred Reporting Items for Systematic Reviews and Meta-Analyses (PRISMA) flow chart.

The literature search (Fig. 1) returned 7097 articles (5025 after removal of duplicate records). After excluding clearly ineligible papers, 328 full-text articles were assessed for eligibility, with 257 of these excluded, most commonly because of timing of lithium initiation (106 articles). In total, 71 articles (Supplementary Data 5), describing 71 studies, met the full eligibility criteria and were included in the literature review, with 30 of these suitable for meta-analysis.

### Study characteristics

Summarised study characteristics can be found Table 1 and full details of individual studies in Supplementary Data 4.1(a–c). In total, 30 542 participants were included across all studies (range: 10–5089, median: 35). Most participants were in the two-arm NRT group (24 052 participants), which consisted of several register studies. Most studies were conducted in out-patient services only (46.5%). The majority of study designs were either RCTs (36.6%) or open label lithium studies (36.6%), 9.9% were retrospective/register studies and 12.7% were naturalistic studies, where patients were followed in clinic. Mean study length was 13.9 months overall (median: 2 months), and two-arm studies had longer study lengths compared with the other two groups (mean: 39.6 months). Most studies (77.5%) used the DSM to assess bipolar disorder. The mean number of participants at baseline for lithium groups was 267, with most people in the two-arm NRT group (*n* = 960). Overall, about a third of participants discontinued the study before they reached the end-point (30.9% and 38.2% from lithium groups and whole-study groups, respectively). The highest tolerability issue was people reporting any adverse event/treatment emergent adverse event (AE/TEAE) (43%), with a mean AE/TEAE of 2.2 per participant (Table 2). The mean lithium levels were 0.7 mmol/L or, when reported as a range, 0.32–1.4 mmol/L, and dose was between 450 and 1800 mg/day.

**Table 1:** Characteristics of studies

	Overall results (*N* = 71)		Randomised controlled trials (*n* = 33)		Non-randomised two-arm studies (*n* = 13)		Non-randomised one-arm studies (*n* = 25)	
	*n*	%	*n*	%	*n*	%	*n*	%
Study characteristics								
Location								
North America	28	39.4	14	42.4	4	30.8	10	40.0
Middle America	0	0	2	0	0	0	0	0
South America	10	14.1	1	3.0	0	0	9	36.0
Europe	17	23.9	4	12.1	8	61.5	5	20.0
Africa	3	4.2	3	9.1	0	0	0	0
Asia	9	12.7	7	21.2	1	7.7	1	4.0
Australia	1	1.4	1	3.0	0	0	0	0
Multiple	3	4.2	3	9.1	0	0	0	0
Setting								
Out-patient	33	46.5	15	45.5	5	38.5	13	52.0
In-patient	14	19.7	10	30.3	1	7.7	3	12.0
Community	2	2.8	2	6.1	0	0	0	0
Multiple	17	23.9	6	18.2	5	38.5	6	24.0
Not reported	5	7.0	0	0	2	15.4	3	12.0
Study design								
Randomised controlled trial	26	36.6	26	78.8	0	0	0	0
Cross-over	0	0	0	0	0	0	0	0
Naturalistic studies	9	12.7	0	0	4	30.8	5	20
Retrospective cohort	1	1.4	0	0	1	7.7	0	0
Open-label lithium	26	36.6	5	15.2	3	23.1	18	72
Other/register	7	9.9	0	0	5	38.5	2	8
Multiple	2	2.8	2	6.1	0	0	0	0
Population								
Bipolar disorder type 1	23	32.4	17	51.1	2	15.4	4	16.0
Bipolar disorder type 2	6	8.5	6	18.2	0	0	0	0
Bipolar disorder mixed	38	53.5	8	24.2	11	84.6	19	76.0
Bipolar disorder and other	3	4.2	2	6.1	0	0	1	4.0
Not reported	1	1.4	0	0	0	0	1	4.0
Current episode								
Manic/hypomanic	13	18.3	12	36.4	0	0	1	4.0
Depression	17	23.9	4	12.1	0	0	13	52.0
Euthymic	4	5.6	3	9.1	1	7.7	0	0
Other/mixed	18	25.4	8	24.2	7	53.8	3	12.0
Multiple	13	18.3	5	15.2	3	23.1	5	20.0
Not reported	6	8.5	1	3.0	2	15.4	3	12.0
Diagnostic tool								
DSM	55	77.5	27	81.8	6	46.2	22	88.0
ICD	8	11.3	2	6.1	5	38.5	1	4.0
Other	3	4.2	1	3.0	1	7.7	1	4.0
Multiple	2	2.8	2	6.1	0	0	0	0
Not reported	3	4.2	1	3.0	1	7.7	1	4.0
Participants, total (mean)	30 542 (430.2)		4918 (149.0)		24 052 (1850.2)		1572 (62.9)	
Study length, months	13.9		8.8		39.6		8.3	
Participants characteristics								
Age, years (mean or median when only available)	37.5		37.5		43.4		34.5	
Female, mean %		53.7		50.1		51.9		59.9
Anxiety comorbidity exclusion								
Yes	22	31.0	7	21.2	1	7.7	14	56.0
No	49	69.0	26	78.8	12	92.3	11	44.0
Substance misuse comorbidity exclusion								
Yes	48	67.6	25	75.8	2	15.4	21	84.0
No	23	32.4	8	24.2	11	84.6	4	16.0
Psychosis comorbidity exclusion								
Yes	26	36.6	9	27.3	4	30.8	13	52.0
No	45	63.4	24	72.7	9	69.2	12	48.0
Suicide ideation exclusion								
Yes	15	21.1	11	33.3	0	0	4	16.0
No	56	78.9	22	66.7	13	100.0	21	84.0

**Table 2:** Lithium treatment

	All	Randomised controlled trials	Two-arm non-randomised controlled trials	One-arm non-randomised controlled trials
Participants				
Whole-study number (baseline mean)	428.8	146.0	1850.2	62.9
Lithium group number (baseline mean)	267.0	62.5	960.3	53.8
Duration (months)	13.9	8.8	39.6	8.3
Lithium levels				
Mean, mmol/L	0.7	0.7	0.7	0.6
Range, mmol/L	0.32–1.4	0.6–1.2	0.5–1.0	0.32–1.4
Lithium dose				
Mean, mg/day	804.5	834.5	922.2	715.2
Range, mg/day	450–1800	600–1800	−	450–1500
Tolerability				
AE/TEAE per participant, mean/s.d.	2.2	2.7	0.9	0.9
% participants with any AE/TEAE	50.1	44.7	−	72.0
% discontinuing due to tolerability/adverse events	14.7	14.8	25.6	3.4
% participants with any SAE	6.7	6.7	−	−
Acceptability				
% discontinuation from lithium group	30.9	37.6	34.5	19.6
% discontinuation (whole study)	38.2	38.0	38.3	−

AE/TEAE, adverse events/treatment emergent adverse events; SAE, serious adverse events.

### Characteristics of participants

The mean age of study participants was 37.5 years, with an almost equal division between male and female participants (Table 1). Participants were equally divided between manic/hypomanic episode, depressive episode or mix of both at baseline. Most studies did not exclude participants for anxiety disorder, psychosis or suicide ideation comorbidity; however, around two-thirds did exclude anyone with a current substance use disorder.

### RoB assessment

In total 17 (of 71) studies were assessed as having a low RoB (Supplementary Data 4.4(a)). The main reasons for higher RoB scores were unclear randomisation or blinding details in RCTs, unclear or unbalanced groups for two-arm NRTs and failure to conduct an intent-to-treat analysis for the one-arm studies. In all three groups, most studies had either low or moderate RoB, with the RCTs and one-arm NRTs having the lowest portion of high RoB (21.2% and 20%, respectively). For full details on RoB analysis on all studies, see Supplementary Data 4.4(b–e).

### Binary efficacy results

#### Positive binary response

**Fig. 2:**
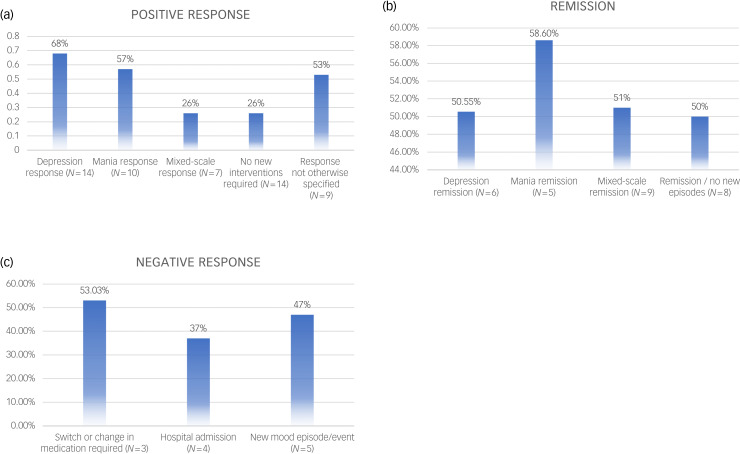
Positive and negative response, and remission results. (a) Positive response results. Depression/mania response: 50% or greater decrease in mood score from baseline to end-point (some variations between studies). Mixed scale response: 50% or greater decrease in both mood scores (mania and depression) from baseline to end-point, or <3 on CGI scale at end-point. Response not otherwise specified: no relapse, 50% or more reduction in time being ill, continuing lithium treatment or response not specified. (b) Remission results. Depression/mania remission: score of 8 or less on the HRSD or YMRS (some variations between studies). Mixed scale remission: score of 8 or less on the HRSD and YMRS (some variation between studies), or <3 on CGI scale continued 2 months after treatment period. (c) Negative response results. CGI, Clinical Global Impression scale; HRSD, Hamilton Rating Scale for Depression; YMRS, Young Mania Rating Scale.

Lithium was reportedly effective at both improving depression and mania (*n* = 37 studies) (Fig. 2(a)), with around two-thirds of participants improving their depression or mania scores by more than 50% (depression: 68%, *n* = 453, 14 studies; mania: 57%, *n* = 548, ten studies). Studies requiring a response on both scales found a much lower response rate (26%, *n* = 338, seven studies); however, to reach this response criteria, both depression and mania scores had to have improved by 50% independent from severity at baseline, which in most cases was either mostly depressed or mostly manic. Where response was defined as not requiring any further medical treatment, the response rate was 26% (*n* = 5078, 14 studies). About half of participants (53%) had a differently defined response to lithium treatment, such as no further relapse or 50% reduction in time being ill (*n* = 1653, nine studies).

#### Remission

Remission (Fig. 2(b)) (*n* = 17 studies) was generally defined as staying well throughout the study or to a set end time of observation, with various similar portraits of euthymia defined using commonly employed scoring thresholds on severity measures, such as scores of ≤8 on the YMRS (see Supplementary Data 4.5 for individual study thresholds). Lithium was shown to be most effective in the remission of manic symptoms (58.6%, *n* = 435, five studies); however, there was a 50% or higher remission rate across all remission categories (depression: 50.55%, *n* = 160, six studies; mixed scale: 51%, *n* = 386, nine studies; other remission: 50%, *n* = 647, eight studies). Average duration of the studies that examined remission was <2 months.

#### Negative binary response

Some (ten studies) (Fig. 2(c)) defined a negative outcome when evaluating the effect of lithium. They found that about half required a switch or change in medication to get better/stay well (53%, *n* = 3155, three studies) or experienced new mood episodes (47%, *n* = 1969, five studies), and about a third of participants receiving lithium treatment were admitted to hospital for their illness (36.7%, *n* = 7230, four studies). Participants were followed up to 12 years, with only three studies with a shorter than 1-year follow-up. The ‘negative binary response’ studies followed the participants on average nearly 3 years (35.1 months).

All individual study results are included in Supplementary Data 4.5.

### Continuous efficacy results (meta-analysis)

**Fig. 3:**
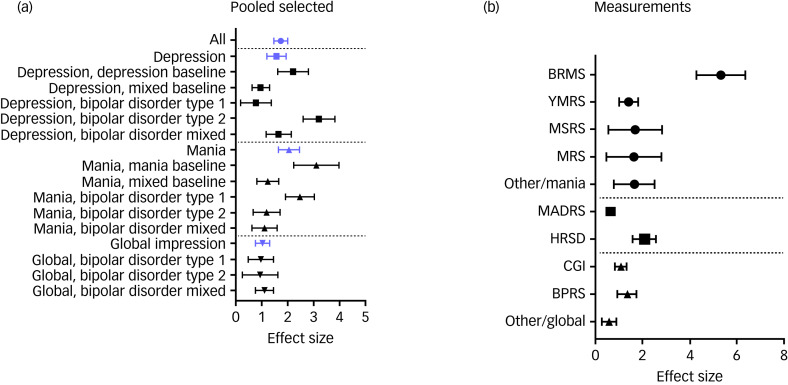
Forest plots of meta-analysis and measurements. (a) Forest plot of relevant meta-analysis. (b) Forest plot of measurements. BPRS, Brief Psychiatric Rating Scale; BRMS, Bech–Rafaelsen Mania Rating Scale; CGI, Clinical Global Impression scale; HRSD, Hamilton Rating Scale for Depression; MADRS, Montgomery–Åsberg Depression Rating Scale; MRS, Mania Rating Scale; MSRS/Beigel, Manic State Rating Scale/Beigel scale; YMRS, Young Mania Rating Scale. In (a), blue denotes category-level effects; squares denote depression effects; point-up triangles denote mania effects; point-down triangles denote global impression effects. In (b), squares denote depression effects; circles denote mania effects; triangles denote global impression effects.

Here, we report first the primary outcome meta-analysis. On measures of manic symptom severity, a pooled Hedges’ *g* of 1.85 was identified (23 studies, 95% CI 1.46–2.25, *I*^2^ = 94%). For depression, Hedge's *g* effect size was 1.56 (18 studies, 95% CI 1.19–1.94, *I*^2^ = 90%) and global illness impression effect size was 0.99 (14 studies, 95% CI 0.73–1.25, *I*^2^ = 87%) (Table 3). The Hedge's *g* effect size of lithium treatment in all studies, independent of outcome measures, was 1.66 (30 studies, 95% CI 1.4–1.93, *I*^2^ = 92%) (Fig. 3(a)).

**Table 3:** Meta-analysis of primary outcome

	Depression						Mania						Global impression					
Subgroup	*k*	*n*	Effect size	s.e.	95% CI	*I*^2^	*k*	*n*	Effect size	s.e.	95% CI	*I*^2^	*k*	*n*	Effect size	s.e.	95% CI	*I*^2^
All	18	507	1.56	0.19	1.19–1.94	90%	23	814	1.85	0.20	1.46–2.25	94%	14	516	0.99	0.13	0.73–1.25	87%
RCT versus NRT																		
RCT	6	307	0.84	0.18	0.49–1.19	86%	16	688	1.49	0.20	1.10–1.98	94%	10	399	0.94	0.13	0.69–1.19	81%
NRT	12	200	1.94	0.27	1.42–2.46	79%	7	126	3.47	0.98	1.56–5.38	95%	4	117	1.18	0.44	0.32–2.04	94%
Baseline affective state																		
Depressed	9	173	2.07	0.30	1.49–2.66	79%	11	295	2.97	0.43	2.13–3.81	95%						
Manic	9	326	0.91	0.17	0.59–1.24	82%	10	446	1.19	0.20	0.80–1.58	90.0%						
Mixed																		
Duration																		
Low (<2 months)	9	232	1.60	0.30	1.01–2.20	91%	13	344	2.38	0.36	1.68–3.07	96%						
Moderate (2–6 months)	8	267	1.68	0.34	1.02–2.35	91%	8	418	1.24	0.20	0.85–1.63	87%						
High (>6 months)	1	8	0.7	0.36	0.0–1.41	0.0%	2	52	1.15	1.23	−1.3 to 3.56	92%						
Bipolar disorder type																		
Bipolar disorder type 1 only	3	94	0.72	0.26	0.20–1.23	68%	14	520	2.22	0.29	1.66–2.78	95%	7	220	0.93	0.24	0.46–1.4	92%
Bipolar disorder type 2 only	2	52	3.07	0.30	2.49–3.66	0.0%	1	12	1.10	0.25	0.06–1.59	0.0%	1	12	0.86	0.32	0.23–1.49	0.0%
Studies including a mixture	12	382	1.55	0.24	1.09–2.01	87%	8	310	1.07	0.24	0.60–1.54	86%	6	284	1.06	0.17	0.73–1.39	83%

*k* indicates the number of studies; *n* indicates the number of participants combined in included studies; *I*^2^ shows heterogeneity. RCT, randomised controlled trial; NRT, non-randomised controlled trial.

### Subgroup meta-analyses

As secondary meta-analysis, we explored relevant subgroups as below (Table 3).

#### Baseline affective state

When analysing results based on manic, depressed or mixed state at baseline, the highest effect size was for mania (effect size 2.97, 11 studies, 95% CI 2.13–3.81, *I*^2^ = 95%), followed by depression (effect size 2.07, nine studies, 95% CI 1.49–2.66, *I*^2^ = 79%). The effect size was higher for mania outcome than depression outcome for the mixed-state group (effect size 1.19, ten studies, 95% CI 0.8–1.58, *I*^2^ = 90%).

#### Bipolar disorder subtype

The highest effect size was seen in ‘bipolar disorder type 2 only’ for depression outcome, when analysing based on bipolar disorder subtype (effect size 3.07, two studies, 95% CI 2.49–3.66, *I*^2^ = 0%), followed by ‘bipolar disorder type 1 only’ for mania outcome (effect size 2.22, 14 studies, 95% CI 1.66–2.78, *I*^2^ = 95%). In studies including a mixture of bipolar disorder subtypes, there were similar effect sizes across the three outcome measures of depression (effect size 1.55, 12 studies, 95% CI 1.09–2.01, *I*^2^ = 87%), mania (effect size 1.07, eight studies, 95% CI 0.6–1.54, *I*^2^ = 86%) and global impression (effect size 1.06, six studies, 95% CI 0.73–1.39, *I*^2^ = 83%).

#### Treatment duration

For mania outcome, the highest effect size was found in studies of 2 months or shorter (effect size 2.38, 13 studies, 95% CI 1.68–3.07, *I*^2^ = 96%), whereas for depression outcome, there was similar response for both studies of 2 months or shorter and in studies of 2–6 months (≤2 months: effect size 1.6, nine studies, 95% CI 1.01–2.20, *I*^2^ = 91%; 2–6 months: effect size 1.68, eight studies, 95% CI 1.02–2.35, *I*^2^ = 87%).

#### Trial design

NRTs had higher effect sizes than RCTs for all outcome measures, with the highest effect size seen for mania outcome (effect size 3.47, seven studies, 95% CI 1.56–5.38, *I*^2^ = 95%) and depression outcome (effect size 1.94, 12 studies, 95% CI 1.42–2.46, *I*^2^ = 79%). Throughout all the analyses, the highest effect size for global impression outcome was seen for the NRT group (effect size 1.18, four studies, 95% CI 0.32–2.04, *I*^2^ = 94%).

We also conducted an analysis using only low and moderate RoB studies (Supplementary Data 4.3); however, the overall results for depression, manic or global impression effect did not change substantially.

For the individual measurements used to assess lithium efficacy, the Bech–Rafaelsen Mania Rating Scale (BRMS) had by far the highest effect size (effect size 5.09, three studies, 95% CI 3.89–6.30, *I*^2^ = 55%), followed by the HRSD (effect size 1.95, 14 studies, 95% CI 1.48–2.43, *I*^2^ = 77%), Manic State Rating Scale (effect size 1.54, three studies, 95% CI 0.53–2.55, *I*^2^ = 75%) and CGI (effect size 1.33, 14 studies, 95% CI 0.99–1.66, *I*^2^ = 90%) (Fig. 3(b), Table 4).

**Table 4:** Meta-analysis of individual measurements

	Depression						Mania						Global impression					
Subgroup	*k*	*n*	Effect size	s.e.	95% CI	*I*^2^	*k*	*n*	Effect size	s.e.	95% CI	*I*^2^	*k*	*n*	Effect size	s.e.	95% CI	*I*^2^
BRMS							3	48	5.09	0.61	3.89–6.30	55%						
YMRS							14	572	1.27	0.19	0.91–1.64	91%						
HRSD	14	224	1.95	0.24	1.48–2.43	77%												
MSRS/Beigel							3	34	1.54	0.51	0.53–2.55	75%						
CGI													14	417	1.33	0.17	0.99–1.66	90%
BPRS													3	101	1.31	0.21	0.91–1.71	43%
MADRS	3	230	0.65	0.07	0.51–0.79	0.0%												
MRS							3	74	1.00	0.59	−0.2 to 2.16	92%						
Other							1	12	1.53	0.41	0.73–2.34	0.0%	1	53	0.58	0.15	0.29–0.87	0.0%

*k* indicates the number of studies; *n* indicates the number of participants combined in included studies; *I*^2^ shows heterogeneity. BRMS, Bech–Rafaelsen Mania Rating Scale; YMRS, Young Mania Rating Scale; HRSD, Hamilton Rating Scale for Depression; MSRS/Beigel, Manic State Rating Scale/Beigel scale; CGI, Clinical Global Impression scale; BPRS, Brief Psychiatric Rating Scale; MADRS, Montgomery–Åsberg Depression Rating Scale; MRS, Mania Rating Scale.

A few articles reported continuous results, but were not suitable for the meta-analysis for reasons such as depression outcomes being reported for patients who were manic at baseline, or more rarely used outcomes such as time to discontinuation of lithium or time to new intervention. All lithium efficacy results are included in Supplementary Data 4.2(a–c).

## Discussion

This is a comprehensive systematic review and meta-analysis of lithium efficacy studies in bipolar disorder spanning several decades, including 71 different studies, totalling 30 542 participants. The included studies employed a diverse range of measures and reported outcomes, such as continuous assessments of symptom amelioration, treatment response, remission, hospital admission, etc.

### Binary outcome data

Lithium was found to be effective in reducing manic and depressive symptoms by more than 50% from baseline in around two-thirds of participants, which was a frequently reported study outcome (31 studies reported depression response, mania response or mixed response as measures of amelioration of symptoms). Lithium appeared to enable 26% of examined participants to remain well without further interventions being required; however, the results in this group varied from 4 to 82%, and follow-up duration varied extensively (2 weeks to 10 years). The negative outcome of ‘intervention being required’ for patients to remain well was reported in 53% of examined patients; however, two out of three studies in this group, equalling 3102 out of 3155 participants, found that 72% of patients had this response. Taken in the round, these findings collectively indicate that around a third remain well on lithium alone and around two-thirds need further intervention to stay well. For around a third of participants, illness progression resulted in hospital admission. Furthermore, it would appear that around half of patients have recurrent mood episodes when treated with lithium (47%) and half do not (in studies reporting remission, 50% reached remission).

Our findings suggest lithium to be most efficacious in providing remission of manic symptoms, but generally half or more of patients achieved euthymia, regardless of pre-treatment affective state, when treated with lithium within the timeframes of the studies, similar to previously reported results from a meta-analysis that found lithium to be effective in preventing all mood episodes, but particularly mania.^10^

Efficacy data composed of mixed-scale response showed poorer response (26%). Similarly, in the meta-analysis, we found that mixed groups (symptoms of both mania and depression) had lower effect of treatment. These results are supported in the literature, as clear mania–depression episodes are considered good predictors of lithium response.^33^ However, it should be noted that the mixed-scale response (using both depression and mania mood scales) did require 50% lowering of both manic and depressive symptoms in samples that did not necessarily have both manic and depressive symptoms at baseline, and we do see a similar approximate 50% remission in mixed-scale groups, suggesting that although the rate of 50% reduction in both manic and depressive symptoms was not around 50–60% in these groups, around 50% did reach remission, similar to the mania and depression groups.

Overall, the various methods used to report a response or remission of symptoms roughly dividing patients in equally sized groups of good responders, partial responders and non-responders, aligning with pre-existing reports across the literature:^22^ We found, within the timeframes of the studies, half to two-thirds of patients have a response to lithium, approximately a third do not require augmented treatment, around half have no further episodes when treated or achieve remission to euthymia, and approximately a third are admitted to hospital.

### Continuous data

Although it appears more people see a significant change in their depressive symptoms (decline of 50% or more on mood scales), the biggest mean change in mood symptoms is for manic symptoms. As presented in Table 4, lithium response effect sizes were largely consistent across measures of manic symptoms, with the notable exception of the BRMS, which by far had the largest effect size (effect size 5.09). Although it is possible that the BRMS is more sensitive to manic symptom changes, two of the three studies to use it recruited a group of patients with bipolar disorder with a very high baseline mania score (means of over 29, which corresponds to ‘marked/severe mania’), therefore allowing for a much greater improvement from baseline when compared with other measures. Furthermore, the analysis only included three studies (*n* = 48), which is a much smaller sample size compared with the YMRS analysis (14 studies, *n* = 572).

In terms of depression response measurements, only two tools were used in a sufficient number of studies to be meta-analysed. The HRSD had a higher effect size (effect size 1.95) compared with the MADRS (effect size 0.65). Both measures were used in a very similar number of participants (*n* = 224 for the HRSD and *n* = 230 for the MADRS), meaning it seems unlikely that sample size differences would account for the difference in observed effect sizes despite it being split over 14 individual studies for the HRSD but only three studies for the MADRS. The explanation for this difference in effect size may lie in our grouping of subversions of the HRSD into one group. Although the MADRS is a unified, 11-item rating scale, we grouped all versions of the HRSD together into one group, meaning that many of these studies were using version of the HRSD that has 17–25 items, potentially allowing for a much greater sensitivity in detecting change from baseline.

As shown in Supplementary Data 4.4(a), RoB ratings showed high variability across included studies. Just over half of the included studies (53.5%) were judged to have a moderate RoB, with the remaining studies split almost equally between low and high RoB (23.9% and 22.5%, respectively). A closer inspection of the data reveals that the low RoB studies were primarily the RCTs of lithium, and the two-arm studies routinely received high RoB ratings (30.8%) (Supplementary Data 4.4(c–e)). There were many registry studies in this group that did not create ‘balanced groups’ and did not ensure equal treatment in various groups, instead having a more naturalistic approach to collecting data, resulting in bias risk. As one would expect, lithium efficacy effect sizes were lower, although still encouraging, across measures of mania, depression and global impression for RCTs when compared with NRTs (see Table 3), given the effort to avoid bias in the results (blinding, randomisation, etc.). Further, meta-analysis of the primary outcome using only studies with a low or moderate RoB returned a good effect size for the treatment of mania (effect size 1.78) and depression (effect size 1.38) (Supplementary Data 4.3). Overall, we believe that although a significant portion of published studies on the efficacy of lithium have a high RoB (22.5%), there is enough quality of evidence returning a good effect size among studies with a low or moderate RoB to recommend lithium as an efficacious treatment for treating depression, mania and mixed states.

### Implications

Lithium has been previously assessed according to GRADE (Grading of Recommendations Assessment, Development and Evaluation) criteria.^34^ Our present findings build upon these, demonstrating, in the largest and first specific systematic review, that across various study designs and response criteria (e.g. improvement of mood symptoms, achieving remission and avoiding hospital admission), around half to two-thirds respond to lithium treatment, and around half of those respond without further treatment needed. The present manuscript is, to our knowledge, the largest review and meta-analysis of lithium efficacy in bipolar disorder in the literature to date, evaluating over 30 000 participants. Our key finding is that lithium is effective in improving bipolar symptoms on measures of depression, mania, and global functioning regardless of baseline mood state. These findings may be of value in encouraging lithium prescription for bipolar disorder when not contraindicated, especially when also considering previous research supporting lithium efficacy compared with other available treatment options.^14^

### Meta-analytic approach

Between-group comparisons of lithium compared with other interventions have been examined in several (recent) meta-analyses and are well established. Our aim instead was to compare efficacy estimates for the different clinical outcome measures used in clinical research as a whole. Traditional between-participants analyses would not have permitted the inclusion of either single-arm trials or multi-arm trials without a common comparator (e.g. placebo). The exclusion of relevant data here would have increased the bias and reduced the scope of comparisons that we could make. Although in general, within-participant (pre–post) meta-analyses are criticised for their limited ability to compare between arms, we argue that they confer a variety of advantages. First, they do not require a common control, which allowed the inclusion of any study with a pre- and post- measure of the outcome. Second, pre–post effect sizes have good clinical face validity because they more closely reflect the size of treatment effects observed naturally (incorporating both those specific to the intervention and non-specific effects over time). Because of this, we highlight that the within-participant effect sizes reported are not to be compared in magnitude to those reported elsewhere reflecting between-participants effects. We note that, conversely, including this range of studies likely increased the between-study heterogeneity within our analyses (see section ‘Limitations’). As well as maximising the literature that could be examined, within-participant meta-analyses also permitted us to indirectly compare effects between analyses, through observing the overlap of confidence intervals (e.g. Strawbridge et al^35^).

### Limitations

Although our review was systematic and comprehensive, the risk of missing eligible papers remains. This includes both in terms of newly published papers that could be eligible and papers that, although relevant, did not meet our inclusion criteria. Indeed, we saw a high exclusion rate for papers that did not include a baseline assessment of bipolar disorder mood state, and therefore were ineligible according to our criteria. Additionally, the scope of the present review is further limited by its exclusion of articles written in languages other than English, and the findings must be viewed in this context. This could be addressed in future research, in addition to comparing the efficacy of lithium with other treatments suitable for bipolar disorder.

The broad inclusion criteria in this review is both a strength and limitation. It was an anticipated limitation that there would not be distinct homogeneity of study design, participants or treatment characteristics between studies, as the aim of the present review was to combine and draw conclusions from the broad clinical research literature, using broad inclusion criteria. The meta-analysis attempted to adjust for these variations to reduce between-study heterogeneity. Despite this, heterogeneity within subanalyses frequently remained high. For the binary data analysis, we would also have expected high heterogeneity had we explored this further; however, this was beyond the scope of this review and is considered an additional limitation. The high heterogeneity may be partly attributed to factors that we were unable to explore in these analyses, such as lithium treatment durations which, as explored further below, can greatly affect the results. However, despite the overall high heterogeneity between studies, the results present with a homogeneous result of around half to two-thirds positive treatment outcome across various efficacy measurements. This heterogeneity should be considered for clinical interpretations of our results, although statistical and clinical heterogeneity are common at both individual and group levels, and we emphasise that our results align well with previous reports.^22^

Furthermore, looking at an effect of any treatment, the study design will affect the results, and it matters what the baseline severity was, how long the patients were treated for and followed up, as well as any subgroup of illness (bipolar disorder type 1 versus type 2, etc.). A diverse range of participant mood presentations were included at baseline, and those in manic or hypomanic (*n* = 13), depressed (*n* = 17) or euthymic (*n* = 4), multiple (*n* = 13) or other/mixed (*n* = 18) presentations were all included in the present review. Given the fluctuating nature of bipolar disorder, as well as the lifelong duration of illness, the study duration and established end-point of data gathering (which varied between 2 weeks and 144 months) matters greatly, as any research will only portrait a limited duration of the bipolar disorder lifespan, and thus any treatment response can only reflect this window in time. To support this, in the meta-analysis we found higher effect sizes in short-term studies (shorter than 2 months) compared with longer studies (longer than 6 months) for both mania (effect size 2.38 *v*. 1.15) and depression outcomes (effect size 1.6 *v*. 0.7); similarly, the studies that found high hospital admission rates (37%) followed patients for 2–12 years. This is a considerable limitation when concluding any long-term effect of lithium in the general bipolar population, and should be considered when viewing our results. It is also worth noting the discontinuation rates (30.9% and 38.2% for lithium groups and whole-study groups, respectively), high burden of side-effects (50.1%) and discontinuation owing to side-effects (14.7%), appearing to align with previous research reporting high discontinuation rates of lithium treatment.^8^ Many of the participants withdrawing from the studies will not be included in the final results of response or remission, and our results do not reflect intolerability as a measure of negative outcome. Although we did a separate meta-analysis for only low and moderate RoB studies and this did not yield substantial different results, this was not done for the binary results, and discontinuation or intolerability might not have been reflected.

Small deviations from the preregistered PROSPERO protocol were made because the present project was expanded to include a meta-analysis. These changes primarily concerned the inclusion of additional study designs from those planned, and the use of the Cochrane RoB tool for all included studies. Because of the value of the meta-analysis and subgroup analyses, it was beyond the scope of the present review to provide a full narrative synthesis of the binary data, as guided by the SWiM (Synthesis without Meta-Analysis) guidelines,^36^ as intended. For transparency, details of all changes made to our preregistration are presented in Supplementary Data 4.6.

Furthermore, the participants examined in included studies may not have been representative of all people with bipolar disorder (e.g. frequent exclusion of substance use disorder or participants under 18 years of age); there was very small sample size of some meta-analysis subgroups; and, although ITT has been included in our RoB tool, the high drop-out rate or side-effects should be considered when evaluating the effect of treatment, as it is likely a large group of people in the studies did not get included in the final efficacy analysis. The short length of lithium treatment and follow-up can equally give an inaccurate portrait of actual efficacy, either not giving patients efficient time to show adequate changes for a response or not following patients consistently through several phases of their lives so as to test the durability of the treatment. And finally, had we had the space to do a thorough subgroup analysis of the binary data, we might have been able to give a more nuanced portrait of lithium treatment efficacy similar to how we reported the meta-analysis results. Additionally, although we expected to include papers that used the Alda scale in our paper, no studies meeting our inclusion criteria were found. Given the Alda scale is a widely used tool in the retrospective assessment of lithium treatment efficacy, it would be interesting to conduct a supplementary search and analysis of those studies that used the Alda scale and compare with the results presented here. Likewise, other outcomes where lithium might be of benefit, such as suicide or cognition, would be useful to include in future studies. Finally, as our study focused solely on the effect of lithium treatment and the different measurements of this and how they compare between them, we did not look at how lithium compares to other available treatments. It would be interesting to do a comparison between lithium and other treatments, including no treatment, on efficacy measurements, but this was beyond the scope of this review.

In summary, it has been known for some time that lithium is effective as both an antimanic and antidepressive agent and our results conclude that lithium is effective in the treatment of bipolar disorder across baseline mood scores, bipolar disorder subtype and length of treatment. However, the present review established that the way any such effect is measured in research varies, and therefore so does the reported effect itself. There is also great variety in how research defines response, e.g. complete remission (>50%) or no further intervention required (26%). This is as much a philosophical question as a scientific one, and so being clear in what is considered a ‘response’ is important when evaluating lithium treatment for clinicians, patients and researchers. Overall, however, all results support around half to two-thirds of patients receiving lithium having a good treatment outcome, around a third staying well with no further treatment needed and around a third to half experience continuous mood episodes or require hospital admission, which seems to be universal regardless of how a response is defined and aligns with previous findings in the literature.

Given the overall good response and remission results across multiple bipolar disorder subgroups, mood states and severity, our findings support lithium's classification as the gold standard of treatment for bipolar disorder.

## Data Availability

The data that support the findings of this study are available on request from the corresponding author, A.H.Y.

## Supplementary material

For supplementary material accompanying this paper visit http://doi.org/10.1192/bjo.2023.64. click here to view supplementary material
