# Comparing measurements of lithium treatment efficacy in people with bipolar disorder: systematic review and meta-analysis – CORRIGENDUM

**DOI:** 10.1192/bjo.2024.807

**Published:** 2024-11-13

**Authors:** Andrea Ulrichsen, Elliot Hampsey, Rosie H. Taylor, Romayne Gadelrab, Rebecca Strawbridge, Allan H. Young

This article originally published with errors in figure 1 and tables 1 and 3. The correct figure and tables are included below.

**Fig. 1:**
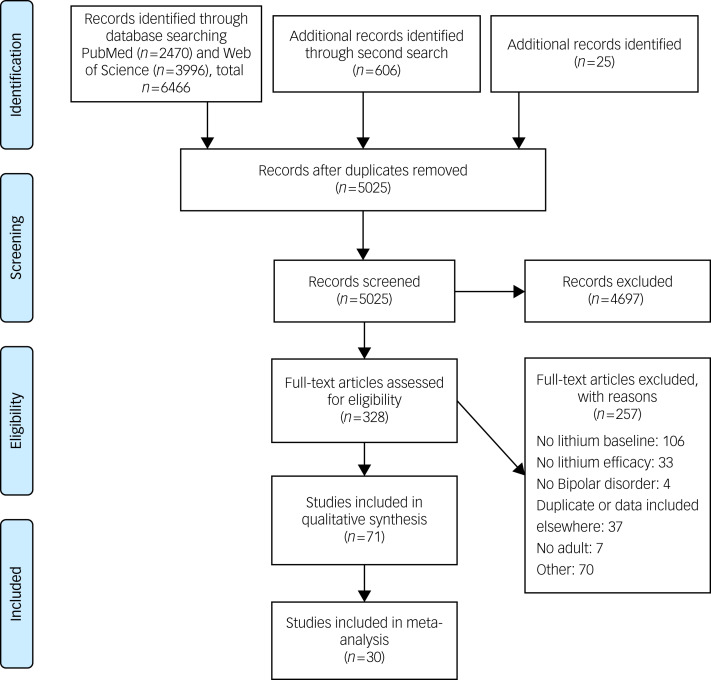
Prisma flow chart

**Table 1:** Characteristics of studies

	Overall results (n = 71)		RCT (n = 33)		Non-randomised 2 arm studies (n = 13)		Non-randomised 1 arm studies (n = 25)	
	*N*	*%*	*N*	*%*	*N*	*%*	*N*	*%*
Study characteristics								
Location								
North America	28	39.4	14	42.4	4	30.8	10	40.0
Middle America	0	0	0	0	0	0	0	0
South America	10	14.1	1	3.0	0	0	9	36.0
Europe	17	23.9	4	12.1	8	61.5	5	20.0
Africa	3	4.2	3	9.1	0	0	0	0
Asia	9	12.7	7	21.2	1	7.7	1	4.0
Australia	1	1.4	1	3.0	0	0	0	0
Multiple	3	4.2	3	9.1	0	0	0	0
Setting								
Outpatient	33	46.5	15	45.5	5	38.5	13	52.0
Inpatient	14	19.7	10	30.3	1	7.7	3	12.0
Community	2	2.8	2	6.1	0	0	0	0
Multiple	17	23.9	6	18.2	5	38.5	6	24.0
n/r	5	7.0	0	0	2	15.4	3	12.0
Study design								
RCT	26	36.6%	26	78.8%	0	0%	0	0%
Cross-over	0	0%	0	0%	0	0%	0	0%
Naturalistic studies	9	12.7%	0	0%	4	30.8%	5	20%
Retrospective cohort	1	1.4%	0	0%	1	7.7%	0	0%
Open label lithium	26	36.6%	5	15.2%	3	23.1%	18	72%
Other/register	7	9.9%	0	0%	5	38.5%	2	8%
Multiple	2	2.8%	2	6.1%	0	0%	0	0%
Population								
BD1	23	32.4	17	51.1	2	15.4	4	16.0
BD2	6	8.5	6	18.2	0	0	0	0
BD mix	38	53.5	8	24.2	11	84.6	19	76.0
BD and other	3	4.2	2	6.1	0	0	1	4.0
n/r	1	1.4	0	0	0	0	1	4.0
Current episode								
Manic/hypomanic	13	18.3	12	36.4	0	0	1	4.0
Depression	17	23.9	4	12.1	0	0	13	52.0
Euthymic	4	5.6	3	9.1	1	7.7	0	0
Other/mix	18	25.4	8	24.2	7	53.8	3	12.0
Multiple	13	18.3	5	15.2	3	23.1	5	20.0
n/r	6	8.5	1	3.0	2	15.4	3	12.0
Diagnostic tool								
DSM	55	77.5	27	81.8	6	46.2	22	88.0
ICD	8	11.3	2	6.1	5	38.5	1	4.0
Other	3	4.2	1	3.0	1	7.7	1	4.0
Multiple	2	2.8	2	6.1	0	0	0	0
n/r	3	4.2	1	3.0	1	7.7	1	4.0
Participants, total (mean)	30542 (430.2)		4918 (149.0)		24052 (1850.2)		1572 (62.9)	
Study length, months	13.9		8.8		39.6		8.3	
Participants characteristics								
Age, years (mean or median when only available)	37.5		37.5		43.4		34.5	
Female, mean %		53.7		50.1		51.9		59.9
Anxiety comorbidity exclusion								
Yes	22	31.0	7	21.2	1	7.7	14	56.0
No	49	69.0	26	78.8	12	92.3	11	44.0
Substance misuse comorbidity exclusion								
Yes	48	67.6	25	75.8	2	15.4	21	84.0
No	23	32.4	8	24.2	11	84.6	4	16.0
Psychosis comorbidity exclusion								
Yes	26	36.6	9	27.3	4	30.8	13	52.0
No	45	63.4	24	72.7	9	69.2	12	48.0
Suicide ideation exclusion								
Yes	15	21.1	11	33.3	0	0	4	16.0
No	56	78.9	22	66.7	13	100.0	21	84.0

N = number of studies; RCT = randomised control studies; BD = bipolar disorder; DSM = Diagnostic and Statistical Manual of Mental Disorders; ICD = International Classification of Diseases

**Table 3:** Meta-analysis of primary outcome

	Depression						Mania						Global impression					
Subgroup	K	N	ES	SE	95% CI	I^2^	k	N	ES	SE	95% CI	I^2^	k	N	ES	SE	95% CI	I^2^
All	18	507	1.56	0.19	1.19–1.94	90%	23	814	1.85	0.20	1.46–2.25	94%	14	516	0.99	0.13	0.73–1.25	87%
RCT vs non-RCT																		
RCT	6	30	0.8	0.1	0.49–1.19	86	16	68	1.49	0.20	1.10–1.98	94%	10	399	0.94	0.13	0.69–1.19	81%
NRT	12	200	1.94	0.27	1.42–2.46	79%	7	126	3.47	0.98	1.56–5.38	95%	4	117	1.18	0.44	0.32–2.04	94%
Baseline affective state																		
Depressed	9	17	2.07	0.30	1.49–2.66	79%												
Manic							11	29	2.97	0.43	2.13–3.81	95%						
Mixed	9	326	0.91	0.17	0.59–1.24	82%	10	446	1.19	0.20	0.80–1.58	90.0%						
Duration																		
Low (<2 months)	9	232	1.60	0.30	1.01–2.20	91%	13	344	2.38	0.36	1.68–3.07	96%						
Moderate (2–6 months)	8	267	1.68	0.34	1.02–2.35	91%	8	418	1.24	0.20	0.85–1.63	87%						
Hight (>6 months)	1	8	0.7	0.36	0.0–1.41	0%	2	52	1.15	1.23	−1.3–3.56	92%						
BD type																		
BD1 only	3	94	0.72	0.26	0.20–1.23	68%	14	520	2.22	0.29	1.66–2.78	95%	7	220	0.93	0.24	0.46–1.4	92%
BD2 only	2	52	3.07	0.30	2.49–3.66	0%	1	12	1.10	0.25	0.06–1.59	0.0%	1	12	0.86	0.32	0.23–1.49	0.0%
Studies including mixture	12	382	1.55	0.24	1.09–2.01	87%	8	310	1.07	0.24	0.60–1.54	86%	6	284	1.06	0.17	0.73–1.39	83%

K = number of studies; N = number of participants combined in included studies; ES = Effect size; SE = Standard error; CI = confidence interval; I^2^ = heterogeneity; RCT = randomised controlled trial; BD = bipolar disorder
