# Supplementary material for: Comparing measurements of lithium treatment efficacy in people with bipolar disorder: systematic review and meta-analysis
Source: BJPsych Open. 2023 May 25;9(3):e98. doi: 10.1192/bjo.2023.64 (PMC10228238; doi:10.1192/bjo.2023.64)
Supplement: Supplementary file 1 [file S2056472423000649sup001.docx]

Supplementary data

Supplementary data 1A: PRISMA 2020 Checklist

| **Section and Topic** | **Item #** | **Checklist item** | **Location where item is reported** |
| --- | --- | --- | --- |
| **TITLE** | | |  |
| Title | 1 | Identify the report as a systematic review. | Page 1 |
| **ABSTRACT** | | |  |
| Abstract | 2 | See the PRISMA 2020 for Abstracts checklist. | See table below, supplementary data 1B |
| **INTRODUCTION** | | |  |
| Rationale | 3 | Describe the rationale for the review in the context of existing knowledge. | Pages 1-2 |
| Objectives | 4 | Provide an explicit statement of the objective(s) or question(s) the review addresses. | Page 2 |
| **METHODS** | | |  |
| Eligibility criteria | 5 | Specify the inclusion and exclusion criteria for the review and how studies were grouped for the syntheses. | Page 3 |
| Information sources | 6 | Specify all databases, registers, websites, organisations, reference lists and other sources searched or consulted to identify studies. Specify the date when each source was last searched or consulted. | Page 3 |
| Search strategy | 7 | Present the full search strategies for all databases, registers and websites, including any filters and limits used. | Page 3 and supplementary dataset 2 |
| Selection process | 8 | Specify the methods used to decide whether a study met the inclusion criteria of the review, including how many reviewers screened each record and each report retrieved, whether they worked independently, and if applicable, details of automation tools used in the process. | Page 3 |
| Data collection process | 9 | Specify the methods used to collect data from reports, including how many reviewers collected data from each report, whether they worked independently, any processes for obtaining or confirming data from study investigators, and if applicable, details of automation tools used in the process. | Page 4 |
| Data items | 10a | List and define all outcomes for which data were sought. Specify whether all results that were compatible with each outcome domain in each study were sought (e.g. for all measures, time points, analyses), and if not, the methods used to decide which results to collect. | Page 4 and tables 1 and 2, and supplementary data 4.1A-C, 4.2A-C, and 4.5 |
|  | 10b | List and define all other variables for which data were sought (e.g. participant and intervention characteristics, funding sources). Describe any assumptions made about any missing or unclear information. | See 10a |
| Study risk of bias assessment | 11 | Specify the methods used to assess risk of bias in the included studies, including details of the tool(s) used, how many reviewers assessed each study and whether they worked independently, and if applicable, details of automation tools used in the process. | Page 4, supplementary data 4.4B-E |
| Effect measures | 12 | Specify for each outcome the effect measure(s) (e.g. risk ratio, mean difference) used in the synthesis or presentation of results. | Pages 4-5 |
| Synthesis methods | 13a | Describe the processes used to decide which studies were eligible for each synthesis (e.g. tabulating the study intervention characteristics and comparing against the planned groups for each synthesis (item #5)). | Pages 4-5 |
|  | 13b | Describe any methods required to prepare the data for presentation or synthesis, such as handling of missing summary statistics, or data conversions. | Pages 4-5 |
|  | 13c | Describe any methods used to tabulate or visually display results of individual studies and syntheses. | Pages 4-5 |
|  | 13d | Describe any methods used to synthesize results and provide a rationale for the choice(s). If meta-analysis was performed, describe the model(s), method(s) to identify the presence and extent of statistical heterogeneity, and software package(s) used. | Pages 4-5 |
|  | 13e | Describe any methods used to explore possible causes of heterogeneity among study results (e.g. subgroup analysis, meta-regression). | Pages 5 |
|  | 13f | Describe any sensitivity analyses conducted to assess robustness of the synthesized results. | N/A |
| Reporting bias assessment | 14 | Describe any methods used to assess risk of bias due to missing results in a synthesis (arising from reporting biases). | Page 4 |
| Certainty assessment | 15 | Describe any methods used to assess certainty (or confidence) in the body of evidence for an outcome. | Page 4 |
| **RESULTS** | | |  |
| Study selection | 16a | Describe the results of the search and selection process, from the number of records identified in the search to the number of studies included in the review, ideally using a flow diagram. | Page 5 and Figure 1 |
|  | 16b | Cite studies that might appear to meet the inclusion criteria, but which were excluded, and explain why they were excluded. | Figure 1 |
| Study characteristics | 17 | Cite each included study and present its characteristics. | Supplementary data 4.1 |
| Risk of bias in studies | 18 | Present assessments of risk of bias for each included study. | Pages 5-6, supplementary data 4.4B-E |
| Results of individual studies | 19 | For all outcomes, present, for each study: (a) summary statistics for each group (where appropriate) and (b) an effect estimate and its precision (e.g. confidence/credible interval), ideally using structured tables or plots. | Supplementary data 4.2A-C, 4.5 |
| Results of syntheses | 20a | For each synthesis, briefly summarise the characteristics and risk of bias among contributing studies. | Pages 5-6, Tables 1, 2, 3A-B, supplementary data 4.4A |
|  | 20b | Present results of all statistical syntheses conducted. If meta-analysis was done, present for each the summary estimate and its precision (e.g. confidence/credible interval) and measures of statistical heterogeneity. If comparing groups, describe the direction of the effect. | Pages 6-7, Figures 2A-C, 3A-B |
|  | 20c | Present results of all investigations of possible causes of heterogeneity among study results. | Pages 6-7, Tables 3A and 3B, Supplementary data 4.3 |
|  | 20d | Present results of all sensitivity analyses conducted to assess the robustness of the synthesized results. | N/A |
| Reporting biases | 21 | Present assessments of risk of bias due to missing results (arising from reporting biases) for each synthesis assessed. | Supplementary data 4.4B-E |
| Certainty of evidence | 22 | Present assessments of certainty (or confidence) in the body of evidence for each outcome assessed. | Supplementary data 4.4A-E |
| **DISCUSSION** | | |  |
| Discussion | 23a | Provide a general interpretation of the results in the context of other evidence. | Pages 7-9 |
|  | 23b | Discuss any limitations of the evidence included in the review. | Pages 10-11 |
|  | 23c | Discuss any limitations of the review processes used. | Pages 10-11 |
|  | 23d | Discuss implications of the results for practice, policy, and future research. | Page 9 |
| **OTHER INFORMATION** | | |  |
| Registration and protocol | 24a | Provide registration information for the review, including register name and registration number, or state that the review was not registered. | Page 3 |
|  | 24b | Indicate where the review protocol can be accessed, or state that a protocol was not prepared. | Page 3 |
|  | 24c | Describe and explain any amendments to information provided at registration or in the protocol. | Page 11, Supp. data 4.6 |
| Support | 25 | Describe sources of financial or non-financial support for the review, and the role of the funders or sponsors in the review. | Page 12 |
| Competing interests | 26 | Declare any competing interests of review authors. | Page 12 |
| Availability of data, code and other materials | 27 | Report which of the following are publicly available and where they can be found: template data collection forms; data extracted from included studies; data used for all analyses; analytic code; any other materials used in the review. | All data and analyses are included in supplementary data. |

*From:*  Page MJ, McKenzie JE, Bossuyt PM, Boutron I, Hoffmann TC, Mulrow CD, et al. The PRISMA 2020 statement: an updated guideline for reporting systematic reviews. BMJ 2021;372:n71. doi: 10.1136/bmj.n71

Supplementary data 1B: PRISMA 2020 for Abstract Checklist

| **Section and Topic** | **Item #** | **Checklist item** | **Reported (Yes/No)** |
| --- | --- | --- | --- |
| **TITLE** | | |  |
| Title | 1 | Identify the report as a systematic review. | Y |
| **BACKGROUND** | | |  |
| Objectives | 2 | Provide an explicit statement of the main objective(s) or question(s) the review addresses. | Y |
| **METHODS** | | |  |
| Eligibility criteria | 3 | Specify the inclusion and exclusion criteria for the review. | Y |
| Information sources | 4 | Specify the information sources (e.g., databases, registers) used to identify studies and the date when each was last searched. | Y |
| Risk of bias | 5 | Specify the methods used to assess risk of bias in the included studies. | Y |
| Synthesis of results | 6 | Specify the methods used to present and synthesise results. | Y |
| **RESULTS** | | |  |
| Included studies | 7 | Give the total number of included studies and participants and summarise relevant characteristics of studies. | Y |
| Synthesis of results | 8 | Present results for main outcomes, preferably indicating the number of included studies and participants for each. If meta-analysis was done, report the summary estimate and confidence/credible interval. If comparing groups, indicate the direction of the effect (i.e. which group is favoured). | Y – as much as space allowed |
| **DISCUSSION** | | |  |
| Limitations of evidence | 9 | Provide a brief summary of the limitations of the evidence included in the review (e.g., study risk of bias, inconsistency and imprecision). | N |
| Interpretation | 10 | Provide a general interpretation of the results and important implications. | Y |
| **OTHER** | | |  |
| Funding | 11 | Specify the primary source of funding for the review. | N |
| Registration | 12 | Provide the register name and registration number. | Y |

From: Page MJ, McKenzie JE, Bossuyt PM, Boutron I, Hoffmann TC, Mulrow CD, et al. The PRISMA 2020 statement: an updated guideline for reporting systematic reviews. BMJ 2021;372:n71. doi: 10.1136/bmj.n71

Supplementary data 2: Search strings used for various search platforms.

Search PubMed

(((((((bipolar disorder*) OR (bipolar affective disorder*) OR (manic depressive disorder*)) AND (lithium)))) NOT ((meta-analysis) OR review))) AND ((((((individual*) OR people) OR patient*) OR subject*) OR participant*) OR human*)

Field: Title/Abstract

Search Web of Science

(((((((bipolar disorder*) OR (bipolar affective disorder*) OR (manic depressive disorder*)) AND (lithium)))) NOT ((meta-analysis) OR review))) AND ((((((individual*) OR people) OR patient*) OR subject*) OR participant*) OR human*)

Field: Topic (Searches title, abstract, author keywords, and Keywords Plus)

Supplementary data 3: PICOS table (Higgins et al., 2019)

| Population | People with bipolar disorder (any type) |
| --- | --- |
| Intervention | Lithium treatment studies |
| Comparison | Different measures of lithium response (symptom rating scales, clinical scales such as the Alda, time to recurrence/rehospitalisation, etc) |
| Outcome | Treatment response rates |
| Study design | RCTs or naturalistic studies |

Higgins, J. P. T., Thomas, J., Chandler, J., Cumpston, M., Li, T., Page, M. J., & Welch, V. A. (2019). *Cochrane Handbook for Systematic Reviews of Interventions*. John Wiley & Sons.

Supplementary data 4: Additional data or analyses

Supplementary data 4.1A: Study characteristics RCT

| Author | Continent | Setting | Partici-pants, N | Age mean/ median | Female, % | Intervention | Drug comparison | Study length, months | BD pop-ulation | Diagnostic tool | Current episode | Anxiety comor-bidity | Substance comor-bidity | Psycho-sis comorbidity | Suicide ideation |
| --- | --- | --- | --- | --- | --- | --- | --- | --- | --- | --- | --- | --- | --- | --- | --- |
|  | 1=North America, 2=Middle America, 3=South America, 4=Europe, 5=Africa, 6=Asia, 7=Australia | 1=out-patient  2= in-patient  3= com-munity |  |  |  | Different treatment groups |  |  | 1=BD1 only (or specified as manic)  2=BD2 only  3=mix of BD  4=BD and other psychopathology | 1=DSM, 2=ICD, 3=other | 1=manic/hypomanic, 2=depression, 3=euthymic, 4=other/all | 0=Comorbidity excluded  1=comorbidity not excluded | 0=Comorbidity excluded  1=comorbidity not excluded | 0=Comorbidity excluded  1=comorbidity not excluded | 0=Comorbidity excluded  1=comorbidity not excluded |
| T. Suppes et al., 2008 | 1 | 3 | 102 | 36.2 | 57.4 | Lithium; Lamotrigine | Lamotrigine | 4 | 2 | 1 | 2 | 1 | 0 | 0 | 0 |
| Astaneh et al 2012 | 6 | 2 | 60 | n/r | 50 | Lithium control; Lithium + gabapentin | Gabapentin | 1.5 | 1 | 1 | 1 | 1 | 0 | 1 | 1 |
| Pal singh 2008 | 6 | 1, 2 | 50 | 18-50 | 28 | Lithium; Verapamil | Verapamil | 1 | 1 | 2 | 1 | 0 | 0 | 0 | 1 |
| Gao et al., 2018 | 1 | 1 | 42 | 44.7 | 66.6 | Lithium; Quetiapine immediate release | Quetiapine | 4 | 3 | 1 | 4 | 1 | 1 | 1 | 1 |
| Bowden et al., 2003 | 1 | 1 | 302 | 41.9 | 52 | Lithium; Lamotrigine; placebo | Lamotrigine | 19 | 1 | 1 | 1 | 0 | 1 | 1 | 0 |
| Clark et al., 1997 | 5 | 2 | 40 | 18-65 | nr | Lithium; Clozapine | Clonazepam | 1 | 1 | 1 | 1 | 1 | 0 | 1 | 1 |
| Bowden et al., 2005 | 4, 6 | 2 | 302 | 38.8 | 40.8 | Lithium; Quetiapine; placebo | Quetiapine | 3 | 1 | 1 | 1 | 1 | 0 | 1 | 1 |
| Shafti 2017 | 6 | 2 | 23 | 29.8 | 0 | Lithium; Aripiprazole | Aripiprazole | 1 | 1 | n/r | 1 | 1 | 0 | 1 | 0 |
| Denicoff et al., 1997 | 1 | 1 | 52 | 41.3 | 52 | Lithium; Carbamazepine; Lithium + Carbamazepine | Carbamazepine | 36 | 3 | 1 | 4 | 0 | 0 | 1 | 1 |
| Amsterdam et al., 2008 | 1 | 1 | 83 | 36.3 | 60 | Lithium; Venlafaxine | Venlafaxine | 3 | 2 | 1 | 2 | 1 | 0 | 0 | 1 |
| Weisler et al., 2011 | 1, 2, 3, 4, 6 | 1 | 1172 | 38.4 | 57.4 | Lithium; Quetiapine; placebo | Quetiapine | 30 | 1 | 1 | 4 | 0 | 0 | 1 | 0 |
| Hollander et al., 2005 | 1 | 3 | 40 | 40 | 50 | Lithium; placebo | n/a | 2.5 | 3 | 1 | 4 | 1 | 0 | 0 | 0 |
| Amsterdam et al., 2010 | 1 | 1 | 148 | 36.1 | 53.9 | Lithium; Fluoxetine, placebo | Fluoxetine | 11.5 | 2 | 1 | 3 | 1 | 0 | 0 | 1 |
| Shansis et al., 2016 | 3 | 1 | 68 | 38.85 | 82.35 | Lithium; Valproic acid; Carbamazepine | Carbamazepine; Valproic acid | 2 | 3 | 1, 3 | 4 | 1 | 0 | 1 | 0 |
| Shafti et al., 2018 | 6 | 2 | 50 | 28.64 | 0 | Lithium; Carbamazepine | Extended-release Carbamazepine | 0.75 | 1 | 1 | 1 | 0 | 0 | 0 | 0 |
| McNamara et al., 2015 | 1 | 2, 3 | 80 | 17.8 | 50 | Lithium; Quetiapine | Quetiapine | 12 | 1 | 1 | 1, 4 | 1 | 0 | 1 | 1 |
| Strakowski et al, 2016 | 1 | 1, 2 | 68 | 18 | 60 | Lithium; Quetiapine; Healthy controls | Quetiapine | 2 | 1 | 1 | 1 | 1 | 0 | 1 | 1 |
| Young et al., 2017 | 1 | 1, 2 | 224 | 67.6 | 50 | Lithium; Divalproex | Divalproex | 2.25 | 1 | 1 | 4 | 1 | 0 | 1 | 0 |
| Singh et al., 2011 | 6 | 2 | 83 | 28.38 | 0 | Lithium one dose daily; Lithium two doses daily | n/a | 1.38 | 1 | 2 | 1 | 1 | 1 | 1 | 1 |
| Ichim et al., 2000 | 5 | 2 | 3 | 31.9 | 46.67 | Lithium; Lamotrigine | Lamotrigine | 1 | 1 | 1 | 1 | 1 | 0 | 1 | 1 |
| Nierenberg et al., 2013 | 1 | 1 | 283 | 38.7 | 54.6 | Lithium + Optimal treatment; Optimal treatment | n/a | 6 | 3 | 1 | 1, 2 | 1 | 1 | 1 | 0 |
| Greil et al., 1997 | 4 | 1, 2 | 144 | 45 | 50 | Lithium; Carbamazepine | Carbamazepine | 30 | 3 | 1, 2 | 4 | 1 | 0 | 1 | 1 |
| Niufan et al., 2008 | 6 | 1, 2 | 140 | 34 | 49.3 | Lithium; Olanzapine | Olanzapine | 1 | 1 | 1 | 1, 4 | 1 | 1 | 1 | 1 |
| Vestergaard et al., 1998 | 4 | 2 | 91 | 42.13 | 64.83516 | Lithium low dose; Lithium high dose | n/a | 24 | 4 | 1 | 4 | 1 | 1 | 1 | 1 |
| Hartong et al., 2003 | 4 | 1 | 98 | 41.9 | 54.3 | Lithium; Carbamazepine | Carbamazepine | 24 | 3 | 1 | 3 | 1 | 1 | 1 | 1 |
| Li et al., 2008 | 6 | 2 | 154 | 33.6 | 48.1 | Lithium; Quetiapine | Quetiapine | 1 | 1 | 3 | 1 | 1 | 0 | 1 | 1 |
| Segal et al., 1998 | 5 | 2 | 45 | 37.1 | 80 | Lithium; Risperidone, Haloperidol | Risperidone, Haloperidol | 1 | 1 | 1 | 1 | 1 | 0 | 1 | 1 |
| Amsterdam et al 2015 | 1 | 1 | 129 | 43.1 | 47.1 | Lithium; Venlafaxine | Venlafaxine | 9 | 2 | 1 | 2 | 1 | 0 | 0 | 1 |
| Altshuler et al 2017 | 1 | 1 | 142 | 39.5 | 59.2 | Lithium; Sertraline; Lithium + Sertraline | Sertraline | 4 | 2 | 1 | 2 | 1 | 0 | 0 | 1 |
| Simhandl et al 1993 | 4 | 1 | 84 | 42 | 69 | Lithium; Carbamazepine High; Carbamazepine low | Carbamazepine | 24 | 4 | 1 | 1, 2 | 1 | 0 | 1 | 1 |
| Calabrese et al 2003 | 15 countries | 1 | 463 | 43.6 | 60 | Lithium; Lamotrigine 50 mg; Lamotrigine 200 mg; Lamotrigine 400 mg; Placebo | Lamotrigine | 22 | 1 | 1 | 3 | 0 | 1 | 1 | 0 |
| Gao 2020 | 1 | 1 | 112 | n/r | 59.3 | Lithium; Divalproex | Divalproex | 0.5 | 3 | 1 | 1, 2, 3 | 1 | 0 | 1 | 1 |
| Parker G 2021 | 7 | 1 | 41 | 30.8 | 50 | Lithium; Lamotrigine | Lamotrigine | 5 | 2 | 1 | n/r | 0 | 0 | 0 | 0 |

Supplementary data 4.1B: Study characteristics NRT 2 arm studies

| Author | Continent | Setting | Participants, N | Age mean/ median | Female, % | Treatment groups | Drug comparison | Study length, months | BD population | Diagnostic tool | Current episode | Anxiety | Substance | Psychosis | Suicide ideation |
| --- | --- | --- | --- | --- | --- | --- | --- | --- | --- | --- | --- | --- | --- | --- | --- |
|  | 1=North America, 2=Middle America, 3=South America, 4=Europe, 5=Africa, 6=Asia, 7=Australia | 1=out-patient  2= in-patient  3= com-munity |  |  |  | Different treatment groups |  |  | 1=BD1 only (or specified as manic), 2=BD2 only, 3=mix of BD, 4=BD and other psychopathology | 1=DSM, 2=ICD, 3=other | 1=manic/hypomanic, 2=depression, 3=euthymic, 4=other/all | 0=Comorbidity excluded  1=comorbidity not excluded | 0=Comorbidity excluded  1=comorbidity not excluded | 0=Comorbidity excluded  1=comorbidity not excluded | 0=Comorbidity excluded  1=comorbidity not excluded |
| Kessing et al., 2012 | 4 | 1, 2 | 4248 | n/r | 58 | Lithium; Lamotrigine | Lamotrigine | n/r | 3 | 2 | 4 | 1 | 1 | 1 | 1 |
| Dalkilic et al., 2000 | 1 | 2 | 47 | 35.9 | 25 | Lithium; Divalproex | Divalproex | 0.33 | 3 | 1 | 4 | 1 | 1 | 1 | 1 |
| Pae et al., 2008 | 6 | n/r | 45 | 32.7 | 55.6 | Lithium; Valproate; Carbamazepine | Valproate; Carbamazepine | 1.2 | 1 | 1 | n/r | 1 | 1 | 1 | 1 |
| Silverstone et al., 2005 | 1 | n/r | 10 | 30.2 | 80 | Lithium depressed group; Lithium euthymic group | n/a | 0.5 | 3 | 1 | 2, 3 | 0 | 0 | 0 | 1 |
| Hayes et al., 2016 | 4 | 1 | 5089 | 44.9 | 57.1 | Lithium; Valproate; Quetiapine; Olanzapine | Valproate; Olanzapine; Quetiapine | 4.2 | 3 | 2 | 4 | 1 | 1 | 0 | 1 |
| Maj et al., 2002 | 4 | 1 | 116 | 34.1 | 55.17 | Lithium psychosis group; Lithium no psychosis group | n/a | 60 | 3 | 1 | 4 | 1 | 1 | 1 | 1 |
| Altamura et al., 2008 | 4 | 1 | 232 | 51.3 | 55 | Lithium; Valproate; Lamotrigine; Quetiapine | Quetiapine; Valproate; Lamotrigine | 48 | 3 | 1 | 3 | 1 | 1 | 0 | 1 |
| Rucci et al 2002 | 1 | 1 | 175 | 35.1 | 56 | Lithium; Divalproex; Carbamazepine | n/a | 24 | 1 | 3 | 1, 2 | 1 | 0 | 1 | 1 |
| Kessing et al., 2011 | 4 | 1, 2 | 4268 | 49 | 58 | Lithium; Valproate | Valproate | 144 | 3 | 2 | 4 | 1 | 1 | 1 | 1 |
| Kessing et al 2014 | 4 | 1, 2 | 4714 | 48.7 | 57.4 | Early intervention Lithium; late intervention Lithium | n/a | 120 | 3 | 2 | 4 | 1 | 1 | 0 | 1 |
| Bohlken 2020 | 4 | 1 | 4990 | 50.6 | 50.3 | Lithium; Valproate; Quetiapine; Olanzapine; Venlafaxine; Citalopram | Valproate; Quetiapine; Olanzapine; Venlafaxine; Citalopram | 24 | 3 | 2 | 4 | 1 | 1 | 1 | 1 |
| Barbuti, 2021 | 4 | 1 ,2 | 70 | 37.88 | 62.9 | Lithium | n/a | 12 | 3 | 1 | 1, 2, 3, 4 | 1 | 1 | 1 | 1 |
| Burton 2021 | 1 | 1, 2 | 48 | 70.9 | 4 | Lithium; SGA (second generation antipsychotic) | Aripiprazole; Quetiapine; Risperidone; Olanzapine; Lurasidone | 37.1 | 3 | n/r | n/r | 1 | 1 | 0 | 1 |

Supplementary data 4.1C: Study characteristics NRT 1 arm studies

| Author | Continent | Setting | Participants, N | Age mean/ median | Female, % | Treatment groups | Drug comparison | Study length, months | BD population | Diagnostic tool | Current episode | Anxiety | Substance | Psychosis | Suicide ideation |
| --- | --- | --- | --- | --- | --- | --- | --- | --- | --- | --- | --- | --- | --- | --- | --- |
|  | 1=North America, 2=Middle America, 3=South America, 4=Europe, 5=Africa, 6=Asia, 7=Australia | 1=out-patient  2= in-patient  3= com-munity |  |  |  | Different treatment groups |  |  | 1=BD1 only (or specified as manic), 2=BD2 only, 3=mix of BD, 4=BD and other psychopathology | 1=DSM, 2=ICD, 3=other | 1=manic/hypomanic, 2=depression, 3=euthymic, 4=other/all | 0=Comorbidity excluded  1=comorbidity not excluded | 0=Comorbidity excluded  1=comorbidity not excluded | 0=Comorbidity excluded  1=comorbidity not excluded | 0=Comorbidity excluded  1=comorbidity not excluded |
| Licht et al 2001 | 4 | 1 | 148 | 45 | 62 | Lithium | n/a | 24 | 3 | 2 | 4 | 1 | 1 | 1 | 1 |
| Selek et al 2013 | 1 | 1, 2 | 41 | 31.8 | nr | Lithium; Healthy control | n/a | 1 | 1 | 1 | 4 | 0 | 0 | 0 | 1 |
| Machado-Vieira et al., 2015 | 3 | 1 | 24 | 28.9 | 70.8 | Lithium | n/a | 1.5 | 3 | 1 | 2 | 0 | 0 | 0 | 1 |
| Moore et al., 2009 | 1 | 1, 3 | 28 | 33 | 46 | Lithium | n/a | 1 | 3 | 1 | 2, 3 | 0 | 0 | 0 | 1 |
| Keck et al., 2001 | 1 | 2 | 15 | 32 | 53 | Lithium | n/a | 0.33 | 1 | 1 | 4 | 1 | 1 | 1 | 1 |
| Machado-Vieira et al., 2016 | 3 | 1 | 23 | 28.2 | 78.3 | Lithium | n/a | 1.5 | 3 | 1 | 2 | 0 | 0 | 0 | 1 |
| Tondo et al., 1997 | 4 | 1 | 86 | 35.4 | 65.1 | Lithium | n/a | 4.5 | 3 | 1 | 1, 2 | 1 | 0 | 1 | 1 |
| Serretti et al., 2004 | 4 | 2 | 83 | 45.6 | 66.3 | Lithium; Lithium plus additional treatment | n/a | 36 | 3 | 1 | n/r | 1 | 0 | 1 | 1 |
| Machado-Vieira et al., 2017 | 3 | 1 | 26 | 28.8 | 80 | Lithium; Healthy control | n/a | 1.5 | 3 | 1 | 2 | 1 | 0 | 1 | 1 |
| Teixeira et al., 2015 | 3 | 1 | 56 | 28.4 | 72.41 | Lithium; Healthy control | n/a | 1.5 | 3 | 1 | 2 | 1 | 0 | 1 | 1 |
| Lowthert et al., 2012 | 1 | n/r | 26 | 38.25 | 70 | Lithium; Healthy control | n/a | 2 | 3 | 1 | 2 | 0 | 0 | 0 | 1 |
| Soeiro-de-Souza et al., 2014 | 3 | n/r | 48 | 28.5 | 76 | Lithium; Healthy control | n/a | 1.5 | 3 | 1 | 2 | 0 | 0 | 0 | 1 |
| Machado-Vieira et al., 2014 | 3 | 1 | 31 | 28.4 | 72.4 | Higher (Li >0.5 mEq/L) and lower (Li <0.5 mEq/L) blood lithium levels | n/a | 1.5 | 3 | 1 | 2 | 0 | 0 | 0 | 1 |
| De Sousa et al., 2015 | 3 | 1 | 49 | 28.4 | 74.1 | Lithium; Healthy control | n/a | 1.5 | 3 | 1 | 2 | 1 | 0 | 1 | 1 |
| Altinay et al., 2017 | 1 | 1, 3 | 41 | 33 | 54 | Lithium; Healthy control | n/a | 2 | 3 | 1 | 1, 2 | 0 | 0 | 0 | 0 |
| Spielberg et al., 2018 | 1 | 1, 3 | 42 | 34.5 | 53.8 | Lithium; Healthy control | n/a | 2 | 3 | 1 | 1, 2 | 0 | 0 | 0 | 0 |
| Breen et al., 2016 | 1 | 1 | 125 | 48.06 | 0 | Lithium | n/a | 3 | 3 | 1 | n/r | 0 | 0 | 0 | 0 |
| Tandon et al., 1981 | 6 | 1, 2 | 50 | 16-65 | 12 | Lithium unipolar; Lithium bipolar | n/a | 1 | 4 | n/r | 1, 2 | 1 | 1 | 1 | 1 |
| Maj et al., 1998 | 4 | 1 | 402 | 40.7 | 55.2 | Lithium | n/a | 60 | 1 | 3 | n/r | 1 | 1 | 1 | 1 |
| Serretti et al., 2000a | 4 | 1 | 61 | 46.7 | 57.4 | Lithium | n/a | 53.5 | 3 | 1 | 2 | 0 | 0 | 0 | 1 |
| De Sousa et al., 2013 | 3 | 1 | 57 | 28.4 | 72.4 | Lithium; Control | n/a | 1.5 | 3 | 1 | 2 | 0 | 0 | 1 | 1 |
| Ananth et al., 2020 | 1 | n/r | 27 | 34 | 47.4 | Lithium | n/a | 2 | n/r | 1 | 2 | 0 | 0 | 0 | 0 |
| Moore et al., 1999 | 1 | 2 | 12 | 36.3 | 58.3 | Lithium | n/a | 1 | 3 | 1 | 2 | 0 | 0 | 0 | 1 |
| Mallinger et al 2008 | 1 | 1, 2 | 45 | 36 | n/r | Lithium | n/a | 0.75 | 1 | 1 | 1 | 1 | 0 | 1 | 1 |
| Machado-Vieira et al., 2016 | 3 | 1 | 26 | 28.8 | 80 | Lithium; Healthy control | n/a | 1.5 | 3 | 1 | 2 | 1 | 0 | 1 | 1 |

Supplementary data 4.2A: Lithium treatment, RCT

| Reference | N (baseline, whole study) | N (baseline, lithium group) | Duration (month) | Dose | Lithium levels | Lithium efficacy response type | Results | Tolerability (Measure / value) | | Acceptability (measure / value) | |
| --- | --- | --- | --- | --- | --- | --- | --- | --- | --- | --- | --- |
| T. Suppes et al., 2008 | 102 | 54 | 4 | 900mg | 0.6–1.2 mEq/L | Depression response | 55.1% | Mean/SD AE/TEAE per participant | mean 9.2 | % Discontinuation from lithium group | 61% |
| Astaneh et al 2012 | 60 | 30 | 1.5 | nr | nr | Change YMRS | -37 points | nr | n/r | N/R | nr |
| Pal singh 2008 | 50 | 25 | 1 | 900mg | 0.8 - 1.2 meq/l | Change YMRS  Change BMRS | -20.84 points  -19.36 points | Mean/SD AE/TEAE per participant | 0.8 | % Discontinuation from lithium group | 0 |
| Gao et al., 2018 | 42 | 18 | 4 | 600 mg/day | 0.5 mmol/L | Time to discontinuation  Change MADRS  Change YMRS  Change HAM-A  Change CGI-S-BP | 7.7 weeks  -11.76  -6.54  -5.73  -2.14 | % discontinuing due to tolerability / AEs | 33.00% | % Discontinuation from lithium group | 77.80% |
| Bowden et al., 2003 | 175 | 46 | 19 | n/r | 0.8-1.1 mEq/L | No response/new episode/event  Time to intervention  Change MRS  Change HAM-D  Change Global assessment scale  Change CGI | 39%  292 days  -0.04  +2.68 (bad)  -3.85  +0.44 (bad) | % discontinuing due to tolerability / AEs | 24% | % Discontinuation from lithium group | 98% |
| Clark et al., 1997 | 40 | 15 | 1 | 750-1800mg | 0.6-1.2 mmol/L | Change CGI  Change MRS  Change GAF Improvement  Change BPRS | -2.66  -20.2  +17.67 (good)  -11.66 | N/R | n/r | % Discontinuation from lithium group | 0 |
| Bowden et al., 2005 | 302 | 98 | 3 | 900 mg/day | 0.8 mEq/L, median at endpoint | Mania response  Mania remission  Change YMRS | 75.5%  72.4%  -20.76 | % any AE/TEAE | 6.10% | % Discontinuation from lithium group | 31.60% |
| Shafti 2017 | 23 | 11 | 1 | 300mg | nr | Change MSRS-I  Change MSRS-F  Change CGI  Change BRMS | -39.06  -27.16  -1.36  -13.67 | N/R | NR | % Discontinuation from lithium group | 36% |
| Denicoff et al., 1997 | 52 | 50 | 36 | 0.5-1.2mmol/l | 0.84 mmol/L | Mixed scale response | 33.3% | % discontinuing due to tolerability / AEs | 3.80% | % Discontinuation from lithium group | 42% |
| Amsterdam et al., 2008 | 83 | 40 | 3 | 966.24mg | 0.64mmol/l | Depression response  Depression remission  Change HAM-D  Change YMRS | 20%  15%  -14  -0.33 | % discontinuing due to tolerability / AEs | 12.50% | % Discontinuation from lithium group | 62.50% |
| Weisler et al., 2011 | 1172 | 364 | 30 | 0.63 mEq/l | 0.6-1.2 meql | No response/no new episode/event | 40% | % discontinuing due to tolerability / AEs | 5.5% | % Discontinuation from lithium group | 51.60% |
| Hollander et al., 2005 | 40 | 18 | 2.5 | 1150 mg (mean) | 0.87 meq/l | Change Mania scale  Change HAM-D | -6.58  -6.67 | % discontinuing due to tolerability / AEs | 0 | % Discontinuation from lithium group | 33.30% |
| Amsterdam et al., 2010 | 148 | 26 | 11.5 | 1027 mg/day | 0.69 mmol/l | Relapse rate | 57.7% | Mean/SD AE/TEAE per participant | 1.58 | % Discontinuation from lithium group | 23.10% |
| Shansis et al., 2016 | 68 |  | 2 | 900-1200 mg/day | 0.8-1.2 mEq/L | Mixed scale response | 20.60%  23.50%  23.5% | n/r | n/r | n/r | n/r |
| Shafti et al., 2018 | 50 | 25 | 0.75 | 965.8 mg/day | 0.74 mEq/L | Change MSRS-F  Change MSRS-I  Change CGI-S  Change CGI-I | -19.69  -10.75  -0.65  -0.96 | N/R | nr | % Discontinuation from lithium group | 24.00% |
| McNamara et al., 2015 | 80 |  | 12 | 600-1800 mg/day | 0.8-1.2 meq/l | Change YMRS (week 8)  Change YMRS (52 weeks) | -17  -17.5 | n/r | n/r | n/r | n/r |
| Strakowski et al, 2016 | 68 |  | 2 | N/R | 0.8-1.2 meq/l | Mixed scale remission  Response NOS | 50%  58% | N/R | N/R | % Discontinuation (whole study) | 38% |
| Young et al., 2017 | 224 | 112 | 2.25 | 300 mg/day | 0.80–0.99 mEq/L | Mania response  Mania remission  Change YMRS | 78.6%  69.6%  -3.9 | % discontinuing due to tolerability / AEs | 22% | % Discontinuation from lithium group | 46.70% |
| Singh et al., 2011 | 83 | 83 | 1.38 | 1149.2 | 0.86 meq/l | Change BRMRS | -27.1 | Side effects tool | n/r | % Discontinuation from lithium group | 25.30% |
| Ichim et al., 2000 | 30 | 15 | 1 | 800mg | 0.743 mmol/L | Mixed scale response  Mania response | 27%  60%  27% | % any SAE | 6.67% | % Discontinuation from lithium group | 20% |
| Nierenberg et al., 2013 | 283 | 141 | 6 | 600 mg/day | 0.47 mEq/L | Mixed scale remission  Change CGI-BP-S  Change MADRS  Change YMRS | 26.5%  -1.22  -8.2  -6.35 | N/R | N/R | % Discontinuation from lithium group | 17.70% |
| Greil et al., 1997 | 144 | 74 | 30 | 27 mmol/day | 0.63 mmol/L | Other response / No intervention required  Other remission / no new episodes | 82%  77% | % any AE/TEAE | 61% | % Discontinuation from lithium group | 18.92% |
| Niufan et al., 2008 | 140 | 71 | 1 | 1110mg (mean) | nr | Mania response  Mania remission  Change CGI-BP-S  Change CGI-BP mania  Change CGI-BP depression  Change YMRS  Change BPRS  Change MADRS | 73.2%  70.4%  -2.22  -2.33  +0.12 (bad)  -20.15  -9.04  -2.51 | % any AE/TEAE | 42.3 | % Discontinuation from lithium group | 21.1 |
| Vestergaard et al., 1998 | 91 | 91 | 24 | 22-28meqv/day | 0.64-0.80 mmol/l | Other response / no intervention required  Other remission / no new episodes | 34%  80% | % discontinuing due to tolerability / AEs | 7.69% | % Discontinuation from lithium group | 66.6% |
| Hartong et al., 2003 | 98 | 44 | 24 | nr | 0.75 mmol/L | Other remission / | 72.7% | % discontinuing due to tolerability / AEs | 11.40% | % Discontinuation from lithium group | 36.30% |
| Li et al., 2008 | 154 | 77 | 1 | 1444.8mg mean | 0.8 mmol/l | Mania response  Mania remission  Mixed scale remission | 59.7%  48.1%  32.5%  48.1% | Mean/SD AE/TEAE per participant | 1.2 | % Discontinuation from lithium group | 19.5% |
| Segal et al., 1998 | 45 | 15 | 1 | 800-1200mg | 0.6-1.2 mmol/L | Change MRS  Change BPRS  Change CGI-S  Change GAF | -12.7  -8.3  -1.3  +22 (good) | Simpson Angus Scale | 0.66 | % Discontinuation from lithium group | 6.70% |
| Amsterdam et al 2015 | 129 | 64 | 9 | 1090 mg/day | 0.72 mEq/L | Depression response  No response / New mood episode/event | 34.4%  26.7% | N/R | n/r | % Discontinuation from lithium group | 11.76% |
| Altshuler et al 2017 | 142 | 49 | 4 | 900 mg/day (minimum) | 0.63 mEq/L | Depression response | 67.4% | % discontinuing due to tolerability / AEs | 29.60% | % Discontinuation from lithium group | 55.10% |
| Simhandl et al 1993 | 84 | 26 | 24 | 450 mg/day | 0.66 mmol/L | Change in hospitalisations  Change duration of episodes  Duration of symptom free interval | -9.4 hospitalisations  -12.1 weeks  +11 weeks (good) | % any AE/TEAE | 69.30% | % Discontinuation from lithium group | 19.23% |
| Calabrese et al 2003 | 463 | 121 | 22 | 900mg (mean) | 0.8 mEq/L | Other response / no intervention required  Time to intervention | 17%  86%  46%  170 days | % discontinuing due to tolerability / AEs | 16% | % Discontinuation from lithium group | 37% |
| Gao 2020 | 112 | 53 | 0.5 | n/r | 0.61 mEq/L. | Mixed scale response  No response / switch/change in medication for response  Change BISS  Change CGI-S  Change CGI-S depression  Change CGI-S mania  Change Q.o.L. | 5.7%  15.1%  -20  -1.28  -1.11  -0.69  +6.04 (good) | Mean/SD AE/TEAE per participant | 0.603774 | % Discontinuation from lithium group | 43% |
| Parker G 2021 | 41 | 20 | 5 | 1070 mg | 0.78 mEq/L | Change HAM-D  Change YMRS  Change CGI-BD  Change CGI-BD mania  Change CGI-BD depression | -10.2  -13  -0.8  -0.9  +0.3 (bad) | % discontinuing due to tolerability / AEs | 11.76% | % Discontinuation from lithium group | 29.41% |

Supplementary data 4.2B: Lithium treatment, NRT 2 arms

| Reference | N (baseline, whole study) | N (baseline, lithium group) | Duration (month) | Dose | Lithium levels | Lithium efficacy response type | Results | Tolerability (Measure / value) | | Acceptability (measure / value) | |
| --- | --- | --- | --- | --- | --- | --- | --- | --- | --- | --- | --- |
| Kessing et al., 2012 | 4248 | 3518 | n/r | n/r | n/r | No response / hospitalisation | 57% | n/r | n/r | % Discontinuation from lithium group | 6.30% |
| Dalkilic et al., 2000 | 47 | 20 | 0.33 | 1,140mg ±471 | 0.64 mEq/L | Length of hospital stay after treatment start | 10.3 days | N/R | n/r | N/R | n/r |
| Pae et al., 2008 | 45 | 30 | 1.2 | 1066.7 mg | N/R | Change YMRS  Change CGI | -27.8  -1.5 | N/R | N/R | N/R | N/R |
| Silverstone et al., 2005 | 10 | 10 | 0.5 | NR | NR | Change HAM-D (depressed group) | -4.4 | N/R | N/R | % Discontinuation from lithium group | 0% |
| Hayes et al., 2016 | 5089 | 1505 | 4.2 | NR | NR | No response / new mood episode/event  Time to treatment failure (75% group) | 75%  2.05 years | n/r | n/r | N/R | n/r |
| Maj et al., 2002 | 116 | 116 | 60 | NR | 0.5-1 mmol/L | Response NOS  Other remission / no new episodes | 56.6%  56.7%  23.3% | N/R | N/R | % Discontinuation from lithium group | 32.70% |
| Altamura et al., 2008 | 232 | 39 | 48 | nr | 0.7 mEq/l | No response / New mood episode/event  Other remission / no new episodes  Euthymia duration | 53.8%  46.2%  33.1 months | N/R | N/R | % Discontinuation from lithium group | 43.6 |
| Rucci et al 2002 | 175 | 166 | 24 | n/r | 0.83 meq/l | Change suicide attempt rate | -94.3 % | % discontinuing due to tolerability / AEs | 9.10% | % Discontinuation (whole study) | 38.30% |
| Kessing et al., 2011 | 4268 | 3549 | 144 | n/r | n/r | No response / Hospitalisation | 54% | N/R | n/r | % Discontinuation from lithium group | 6% |
| Kessing et al 2014 | 4714 | 4714 | 120 | nr | n/r | Other response / No intervention required | 13.3%  13.2%  6.3%  6.7%  8.7%  10.1%  4%  4.2% | N/R | n/r | N/R | n/r |
| Bohlken 2020 | 4990 | 1098 | 24 | NR | n/r | Response NOS | 23.7%  32.9% | N/R | n/r | N/R | n/r |
| Barbuti, 2021 | 70 | 70 | 12 | Baseline 15 mmol/day, endpoint 22 mmol/day | 0.63 mEq/L | Change FAST  Change CGI-BD (prolonged release group)  Change CGI-BD (immediate release group) | -15.81  -1.61  -1.56 | Mean/SD AE/TEAE per participant | 0.878788 | % Discontinuation from lithium group | 53% |
| Burton 2021 | 48 | 24 | 37.1 | 560 mg | n/r | No response / Hospitalisation  Time to discontinuation of treatment (mean) | 14%  1128.7 days | % discontinuing due to tolerability / AEs | 42% | % Discontinuation from lithium group | 100 |

Supplementary data 4.2C: Lithium treatment, NRT 1 arm

| Reference | N (baseline, whole study) | N (baseline, lithium group) | Duration (month) | Dose | Lithium levels | Lithium efficacy response type | Results | Tolerability (Measure / value) | | Acceptability (measure / value) | |
| --- | --- | --- | --- | --- | --- | --- | --- | --- | --- | --- | --- |
| Licht et al 2001 | 148 | 132 | 24 | nr | 0.63 +/- 0.18 mmol/ | No response / hospitalisation  Other response / No intervention required | 21.6%  30.4% | % any AE/TEAE | 72% | % Discontinuation from lithium group | 48.00% |
| Selek et al 2013 | 41 | 30 | 1 | nr | 0.67 mEq/L | Mixed scale response  Mixed scale remission | 50%  25% | N/R | n/r | % Discontinuation from lithium group | 20% |
| Machado-Vieira et al., 2015 | 24 | 24 | 1.5 | 712 mg/day, max | 0.49 mEq/L | Depression response  Depression remission | 91%  71% | N/R | n/r | N/R | n/r |
| Moore et al., 2009 | 28 | 28 | 1 | nr | 0.9 meq/l | Depression response | 39.3% | N/R | n/r | % Discontinuation from lithium group | 0 |
| Keck et al., 2001 | 15 | 15 | 0.33 | 1380 mg/day | 1.0 mEq/L +/- 0.2 | Mania response  Change HAM-D-24  Change YMRS | 60%  -11.2  -16.6 | Mean/SD AE/TEAE per participant | 0.933333 | % Discontinuation from lithium group | 20% |
| Machado-Vieira et al., 2016 | 23 | 23 | 1.5 | 710.05 mg/day, mean | 0.49 mEq/L, endpoint mean | Depression response  Depression remission  Mixed scale remission  Change HAM-D | 85%  65.2%  60%  -15.2 | N/R | n/r | N/R | NR |
| Tondo et al., 1997 | 86 | 86 | 4.5 | nr | 0.62 meq/l | Change episodes per year  Change hospitalisations per year  Change time spent ill | -1.48 episodes  -0.48  -32.5% | N/R | n/r | N/R | n/r |
| Serretti et al., 2004 | 83 | 83 | 36 | mean 1173 mg/die | 0.65–0.75mMol/l | Response NOS | 56.6% | N/R | N/R |  | n/r |
| Machado-Vieira et al., 2017 | 26 | 26 | 1.5 | 671.1mg | 0.48 mmL | Depression response  Mixed scale response  Change HAM-D  Change CGI | 85%  60%  -14.5  -1.85 | n/r | n/r | % Discontinuation from lithium group | 23.10% |
| Teixeira et al., 2015 | 56 | 29 | 1.5 | 450 mg/day | 0.49 mEq/L | Depression response  Mixed scale remission | 82.8%  62.1% | N/R | n/r | N/R | N/R |
| Lowthert et al., 2012 | 26 | 26 | 2 | 0.6-1.2 mEq/L | 0.64 mmol/L | Depression response | 50% | N/R | N/R | % Discontinuation from lithium group | 23.10% |
| Soeiro-de-Souza et al., 2014 | 48 | 25 | 1.5 | 450 mg/day | 0.49 mEq/L | Depression response  Depression remission | 84%  64% | Side effects tool | 28% weight gain | % Discontinuation from lithium group | 0% |
| Machado-Vieira et al., 2014 | 31 | 31 | 1.5 | 450-900 mg/day | 0.49 mEq/L | Depression response  Depression remission | 86.2%  62% | Side effects tool | polydipsia/polyuria (62.1%), cognitive complaints (41.4%), nausea (31.0%), increased oniric activity (31.0%) and sedation (31.0%). | % Discontinuation from lithium group | 6.50% |
| De Sousa et al., 2015 | 49 | 27 | 1.5 | 450 mg/day start | 0.49 mEq/L | Depression response  Mixed scale remission | 85%  63% | N/R | N/R | % Discontinuation from lithium group | 0% |
| Altinay et al., 2017 | 41 | 29 | 2 | 600 mg/day | 0.67 | Change HAM-D (depressed group)  Change HAM-D (hypomanic group)  Change YMRS (depressed group)  Change YMRS (hypomanic group)  Change CGI-S (depressed group)  Change CGI-S (hypomanic group) | -11  +3 (bad)  0  -12.5  -1.3  -0.9 | Side effects tool | headache, increased appetite, dry mouth, blurred vision, dizziness, muscle twitching, bad taste after the drug was taken, heart palpitations, fatigue, nervousness and | % Discontinuation from lithium group | 17.20% |
| Spielberg et al., 2018 | 42 | 29 | 2 | 600 mg/day | 0.7 mEq/L | Change HAM-D (depressed group)  Change HAM-D (manic group)  Change YMRS (depressed group)  Change YMRS (manic group) | -7.7  -0.3  -1.7  -10.2 | N/R | N/R | % Discontinuation from lithium group | 10.30% |
| Breen et al., 2016 | 125 | 125 | 3 | NR | n/r | Response NOS | 50% | N/R | N/R | % Discontinuation from lithium group | 87.20% |
| Tandon et al., 1981 | 50 | 50 | 1 | 600-1500 mg/day | 0.32-1.23 mEq/L | Mania response  Change HAM-D  Change Beigel’s rating scale | 26%  52%  -20.8  -13.3 | N/R | N/R | N/R | N/R |
| Maj et al., 1998 | 402 | 402 | 60 | NR | 0.5-1 mmol/L | Response NOS  Other remission / no new episodes | 61%  85%  23% | % discontinuing due to tolerability / AEs | 6.72 | % Discontinuation from lithium group | 38.60% |
| Serretti et al., 2000a | 61 | 61 | 53.5 | nr | 0.4-0.7 meq/l | Other remission / no new episodes | 21.3%  54.1% | N/R | n/r |  | n/r |
| De Sousa et al., 2013 | 57 | 29 | 1.5 | nr | 0.49 mmol | Depression response  Mixed scale remission  Change HAM-D  Change YMRS | 86.2%  62.1%  -15.3  -2.3 | N/R | n/r | % Discontinuation from lithium group | 3.40% |
| Ananth et al., 2020 | 27 | 27 | 2 | nr | 0.8–1.2 mEq/l | Depression remission  Change HAM-D | 26.3%  -11.8 | N/R | n/r | % Discontinuation from lithium group | 29.63% |
| Moore et al., 1999 | 12 | 12 | 1 | nr | 0.8-1.2 meq/l | Change HAM-D | -6.75 | % discontinuing due to tolerability / AEs | 0 | % Discontinuation from lithium group | 0 |
| Mallinger et al 2008 | 45 | 45 | 0.75 | 900-1200 mg/day | 0.8-1.4 mmol/l | Mania response | 58% | N/R | n/r | % Discontinuation from lithium group | 2.22% |
| Machado-Vieira et al., 2016 | 26 | 26 | 1.5 | 671mg | 0.48 mmol/L | Depression response  Depression remission  Mixed scale remission  Change HAM-D | 85%  62.5%  60%  -15.2 | N/R | n/r | % Discontinuation from lithium group | 23.10% |

Supplementary data 4.3: Meta-analysis of primary outcome, only low and moderate risk of bias studies

| Outcome | Depression | | | | | | Mania | | | | | | Global impression | | | | | |
| --- | --- | --- | --- | --- | --- | --- | --- | --- | --- | --- | --- | --- | --- | --- | --- | --- | --- | --- |
| Subgroup | k | N | ES | SE | 95% CI | I^2^ | k | N | ES | SE | 95% CI | I^2^ | k | N | ES | SE | 95% CI | I^2^ |
| All | 13 | 439 | 1.38 | 0.20 | 0.99-1.77 | 90% | 18 | 739 | 1.78 | 0.22 | 1.34-2.22 | 95% | 12 | 421 | 0.98 | 0.14 | 0.69-1.26 | 89% |
| RCT vs non-RCT  RCT  NRT | 5  8 | 295  144 | 0.57  2.05 | 0.10  0.35 | 0.38-0.76  1.36-2.74 | 56%  82% | 14  4 | 646  93 | 1.55  3.47 | 0.22  1.45 | 1.12-1.99  0.63-6.32 | 95%  67% | 9  3 | 387  105 | 0.95  1.16 | 0.14  0.57 | 0.68-1.21  0.05-2.27 | 84%  96% |
| Baseline affective sate  Depressed  Manic  Mixed | 6  7 | 125  314 | 2.05  0.68 | 0.43  0.13 | 1.21-2.88  0.43-0.93 | 86%  68% | 8  8 | 426  421 | 3.04  1.13 | 0.54  0.22 | 1.99-4.09  0.69-1.56 | 96%  91% |  |  |  |  |  |  |

Supplementary data 4.4A: Overall Risk of Bias results

| TOTAL RoB | |
| --- | --- |
| High | N = 16 (22.5%) |
| Moderate | N = 38 (53.5%) |
| Low | N = 17 (23.9%) |
| **RCT** | |
| High | N = 7 (21.2%) |
| Moderate | N = 13 (39.4%) |
| Low | N = 13 (39.4%) |
| **Non-RCT, 2 arm** | |
| High | N = 4 (30.8%) |
| Moderate | N = 6 (46.2%) |
| Low | N = 3 (23.1%) |
| **Non-RCT, 1 arm** | |
| High | N = 5 (20%) |
| Moderate | N = 19 (76%) |
| Low | N = 1 (4%) |

Supplementary data 4.4B: Risk of bias scoring key

| Random allocation | Allocation concealment | Blinding | Rater blinding | Equal treatment | Balanced groups | Appropriate outcomes | ITT | Deviations |
| --- | --- | --- | --- | --- | --- | --- | --- | --- |
| Were the participants randomised to treatment in a truly random fashion? | Were participants/raters unable to determine what intervention a participant would get until they were assigned? | Were participants and those delivering intervention blinded to assigned intervention? | Were outcome assessors blind to intervention? If not, could this knowledge influence ratings? | Did groups receive the same treatment, including monitoring, follow up type/length etc. | Did treatment groups have similar characteristics? If not, could this confound effect of intervention? | 1) Were groups assessed using different measures? 2) Were several analyses used for same outcome? | Was *everyone* assigned to Li analysed, regardless of whether they completed or adhered? | Did study deviate from plans in design, treatment, or analysis? |
| 1 = yes  0 = no  0.5=for unclear/unstated or method controlled by humans  Non-RCT = N/A | 1 = yes  0 = no  0.5 = unclear, or if it just described as "randomised".  Non-RCT = N/A | 1 = yes to both  0 = no  0.5 = if only one part was blinded.  Non-RCT = N/A | 1 = yes  0 = no  0.5 = if blinded but <5% reported to have been unblinded.  Non-RCT = N/A | 1 = yes, no evidence of different methods in study design between groups.  0 = no  0.5 = minor differences  N/A for one arm trials | 1 = yes  0 = no  0.5 = minor differences  N/A for one arm trials | 1 = no to both  0 = yes to both  0.5 = yes to either/both but justification provided. | 1 = yes, everyone was analysed.  0 = no  0.5 = <5% of patients were excluded (not taking intervention or lack of outcome) and no correction for bias potential in analyses. | 1 = no  0 = yes, evidence of deviation that may have influenced efficacy outcomes  0.5 = no evidence of pre-specified methods or outcomes |

Supplementary data 4.4C: Risk of bias results, RCT

| Authors | Random allocation | Allocation concealment | Blinding (participant & intervention) | Rater blinding | Equal treatment | Balanced groups | Appropriate outcomes | ITT | Deviations | ROB score |
| --- | --- | --- | --- | --- | --- | --- | --- | --- | --- | --- |
| T. Suppes et al., 2008 | 0.5 | 0 | 0 | 1 | 0.5 | 1 | 1 | 0 | 1 | High |
| Astaneh et al 2012 | 0.5 | 0.5 | 0 | 0 | 0.5 | 1 | 1 | 1 | 1 | High |
| Pal singh 2008 | 0.5 | 0.5 | 1 | 1 | 1 | 1 | 1 | 1 | 1 | Low |
| Gao et al., 2018 | 0.5 | 0.5 | 0 | 1 | 1 | 1 | 1 | 1 | 1 | Moderate |
| Bowden et al., 2003 | 0.5 | 0.5 | 1 | 1 | 1 | 1 | 1 | 1 | 1 | Low |
| Clark et al., 1997 | 0.5 | 0.5 | 0 | 0 | 1 | 1 | 1 | 1 | 1 | Moderate |
| Bowden et al., 2005 | 0.5 | 0.5 | 1 | 1 | 1 | 1 | 1 | 1 | 1 | Low |
| Shafti 2017 | 0.5 | 0.5 | 1 | 1 | 1 | 1 | 1 | 1 | 1 | Low |
| Denicoff et al., 1997 | 0.5 | 0.5 | 1 | 0.5 | 1 | 1 | 1 | 0 | 1 | Moderate |
| Amsterdam et al., 2008 | 0.5 | 0.5 | 0 | 0 | 1 | 1 | 1 | 1 | 1 | Moderate |
| Weisler et al., 2011 | 1 | 1 | 1 | NR | 1 | 1 | 1 | 1 | 1 | Low |
| Hollander et al., 2005 | 0.5 | 0.5 | 1 | NR | 1 | 1 | 1 | 0 | 1 | Moderate |
| Amsterdam et al., 2010 | 0.5 | 0.5 | 1 | 1 | 1 | 1 | 1 | 1 | 1 | Low |
| Shansis et al., 2016 | 0.5 | 0.5 | 0 | 0 | 1 | NR | 1 | 1 | 1 | High |
| Shafti et al., 2018 | 0.5 | 0 | 1 | 1 | 1 | 1 | 1 | 0 | 1 | Moderate |
| McNamara et al., 2015 | 0 | 0 | 0 | 0 | 1 | 1 | 1 | 0 | 1 | High |
| Strakowski et al, 2016 | 0 | 0 | 0 | 0 | 1 | 1 | 1 | 0 | 1 | High |
| Young et al., 2017 | 1 | 1 | 1 | 1 | 1 | 1 | 1 | 1 | 1 | Low |
| Singh et al., 2011 | 0.5 | 0 | 0 | 1 | 1 | 1 | 1 | 1 | 1 | Moderate |
| Ichim et al., 2000 | 0.5 | 0.5 | 1 | 1 | 1 | 1 | 1 | 1 | 1 | Low |
| Nierenberg et al., 2013 | 1 | 1 | 0 | 1 | 1 | 1 | 1 | 1 | 1 | Moderate |
| Greil et al., 1997 | 0.5 | 1 | 0 | 0 | 1 | 1 | 1 | 1 | 1 | Moderate |
| Niufan et al., 2008 | 0.5 | 0.5 | 1 | 0 | 1 | 1 | 1 | 1 | 1 | Moderate |
| Vestergaard et al., 1998 | 1 | 0.5 | 0 | 0 | 1 | 1 | 1 | 1 | 1 | Moderate |
| Hartong et al., 2003 | 1 | 1 | 1 | 1 | 1 | 1 | 1 | 0.5 | 0.5 | Low |
| Li et al., 2008 | 0.5 | 0.5 | 1 | 1 | 1 | 1 | 1 | 1 | 1 | Low |
| Segal et al., 1998 | 0.5 | 0.5 | 1 | 1 | 1 | 1 | 1 | 1 | 1 | Low |
| Amsterdam et al 2015 | 1 | 1 | 1 | 1 | 1 | 1 | 1 | 0 | 1 | Moderate |
| Altshuler et al 2017 | 1 | 1 | 1 | 1 | 1 | 1 | 1 | 1 | 1 | Low |
| Simhandl et al 1993 | 0.5 | 0.5 | 0 | 0 | 1 | 1 | 1 | 0 | 1 | High |
| Calabrese et al 2003 | 0.5 | 0.5 | 1 | 1 | 1 | 1 | 1 | 1 | 1 | Low |
| Gao 2020 | 0.5 | 0.5 | 0 | 0 | 1 | 1 | 1 | 1 | 1 | Moderate |
| Parker G 2021 | 0.5 | 0.5 | 0 | 0.5 | 1 | 1 | 1 | 0 | 0 | High |

| Low risk = <1 criteria rated high RoB and <4 unclear RoB |
| --- |
| High risk = >4 criteria rated high or unclear RoB |
| Moderate risk if not meeting criteria for high or low risk of bias. |

Supplementary data 4.4D: Risk of bias results, NRT 2 arm studies

| Authors | Random allocation | Allocation concealment | Blinding (participant & intervention) | Rater blinding | Equal treatment | Balanced groups | Appropriate outcomes | ITT | Deviations | ROB score |
| --- | --- | --- | --- | --- | --- | --- | --- | --- | --- | --- |
| Kessing et al., 2012 | n/a | n/a | n/a | n/a | 0.5 | 0.5 | 1 | 1 | 1 | High |
| Dalkilic et al., 2000 | n/a | n/a | n/a | n/a | 1 | 1 | 1 | 1 | 1 | Low |
| Pae et al., 2008 | n/a | n/a | n/a | n/a | 1 | 1 | 1 | 1 | 1 | Low |
| Silverstone et al., 2005 | n/a | n/a | n/a | n/a | 1 | 0.5 | 1 | 1 | 1 | Moderate |
| Hayes et al., 2016 | n/a | n/a | n/a | n/a | 0 | 0.5 | 1 | 1 | 1 | High |
| Maj et al., 2002 | ? | n/a | n/a | n/a | 1 | 1 | 1 | 0.5 | 1 | Moderate |
| Altamura et al., 2008 | n/a | n/a | n/a | n/a | 1 | 0 | 1 | 0 | 1 | High |
| Rucci et al 2002 | n/a | n/a | n/a | n/a | 1 | n/r | 1 | 1 | 1 | Moderate |
| Kessing et al., 2011 | n/a | n/a | n/a | n/a | 1 | 0.5 | 1 | 1 | 1 | Moderate |
| Kessing et al 2014 | n/a | n/a | n/a | n/a | 0 | 0.5 | 1 | 1 | 1 | High |
| Bohlken 2020 | n/a | n/a | n/a | n/a | 1 | 0.5 | 1 | 1 | 1 | Moderate |
| Barbuti, 2021 | n/a | n/a | n/a | n/a | 1 | 1 | 1 | 0 | 1 | Moderate |
| Burton 2021 | n/a | n/a | n/a | n/a | 1 | 1 | 1 | 1 | 1 | Low |

| Low risk = <1 criteria rated high RoB and <5 unclear RoB |
| --- |
| High risk = >5 criteria rated high or unclear RoB |
| Moderate risk if not meeting criteria for high or low risk of bias. |

Supplementary data 4.4E: Risk of bias results, NRT 1 arm studies

| Authors | Random allocation | Allocation concealment | Blinding (participant & intervention) | Rater blinding | Equal treatment | Balanced groups | Appropriate outcomes | ITT | Deviations | ROB score |
| --- | --- | --- | --- | --- | --- | --- | --- | --- | --- | --- |
| Licht et al 2001 | n/a | n/a | n/a | n/a | n/a | n/a | 1 | 1 | 1 | Moderate |
| Selek et al 2013 | n/a | n/a | n/a | n/a | n/a | n/a | 1 | 1 | 1 | Moderate |
| Machado-Vieira et al., 2015 | n/a | n/a | n/a | n/a | n/a | n/a | 1 | 1 | 1 | Moderate |
| Moore et al., 2009 | n/a | n/a | 0.5 | 1 | n/a | n/a | 1 | 1 | 1 | Low |
| Keck et al., 2001 | n/a | n/a | n/a | n/a | n/a | n/a | 1 | 1 | 1 | Moderate |
| Machado-Vieira et al., 2016 | n/a | n/a | n/a | n/a | n/a | n/a | 1 | 1 | 1 | Moderate |
| Tondo et al., 1997 | n/a | n/a | n/a | n/a | n/a | n/a | 1 | 1 | 1 | Moderate |
| Serretti et al., 2004 | n/a | n/a | n/a | n/a | n/a | n/a | 1 | 1 | 1 | Moderate |
| Machado-Vieira et al., 2017 | n/a | n/a | n/a | n/a | n/a | n/a | 1 | 1 | 1 | Moderate |
| Teixeira et al., 2015 | n/a | n/a | n/a | n/a | n/a | n/a | 1 | 1 | 1 | Moderate |
| Lowthert et al., 2012 | n/a | n/a | n/a | n/a | n/a | n/a | 1 | 1 | 1 | Moderate |
| Soeiro-de-Souza et al., 2014 | n/a | n/a | n/a | n/a | n/a | n/a | 1 | 1 | 1 | Moderate |
| Machado-Vieira et al., 2014 | n/a | n/a | n/a | n/a | n/a | n/a | 1 | 1 | 1 | Moderate |
| De Sousa et al., 2015 | n/a | n/a | n/a | n/a | n/a | n/a | 1 | 1 | 1 | Moderate |
| Altinay et al., 2017 | n/a | n/a | n/a | n/a | n/a | n/a | 1 | 0.5 | 1 | High |
| Spielberg et al., 2018 | n/a | n/a | n/a | n/a | n/a | n/a | 1 | 0.5 | 1 | High |
| Breen et al., 2016 | n/a | n/a | n/a | n/a | n/a | n/a | 1 | 0 | 1 | High |
| Tandon et al., 1981 | n/a | n/a | n/a | n/a | n/a | n/a | 1 | 1 | 1 | Moderate |
| Maj et al., 1998 | n/a | n/a | n/a | n/a | n/a | n/a | 1 | 0 | 1 | High |
| Serretti et al., 2000a | n/a | n/a | n/a | n/a | n/a | n/a | 1 | 1 | 1 | Moderate |
| De Sousa et al., 2013 | n/a | n/a | n/a | n/a | n/a | n/a | 1 | 1 | 1 | Moderate |
| Ananth et al., 2020 | n/a | n/a | n/a | n/a | n/a | n/a | 1 | 1 | 1 | Moderate |
| Moore et al., 1999 | n/a | n/a | n/a | n/a | n/a | n/a | 1 | 1 | 1 | Moderate |
| Mallinger et al 2008 | n/a | n/a | n/a | n/a | n/a | n/a | 1 | 1 | 1 | Moderate |
| Machado-Vieira et al., 2016 | n/a | n/a | n/a | n/a | n/a | n/a | 1 | 0.5 | 1 | High |

| Low risk = <1 criteria rated high RoB and <6 unclear RoB |
| --- |
| High risk = >6 criteria rated high or unclear RoB |
| Moderate risk if not meeting criteria for high or low risk of bias. |

Supplementary data 4.5: Lithium efficacy of binary data

| Lithium response type | Reference | Depression baseline | Mania baseline | N analysed | Measurement | Outcome description | Result |
| --- | --- | --- | --- | --- | --- | --- | --- |
| Depression response | Suppes et al 2008 | HAM-D-17 21.2; MADRS 30.2 | YMRS 6.71 | 49 | HAM-D 17 | Response: 50% or higher decrease in score from baseline to endpoint | 55.10% |
|  | Machado-vieira 2015 | HAM-D 21.7 | YMRS 4.7 | 24 | HAM-D | Response: 50% or higher decrease in score from baseline to endpoint | 91% |
|  | Moore et al 2009 | HAM-D 17.6 | NR | 28 | HAM-D | Response: 50% or higher decrease in score from baseline to endpoint | 39.30% |
|  | Amsterdam et al., 2008 | HAM-D 28.8 | YMRS 1.2 | 40 | HAM-D 28 | Response: 50% or higher decrease in score from baseline to endpoint | 20% |
|  | Machado-Vieira et al., 2017 | HAM-D 22.4 | N/A | 20 | HAM-D | Response: 50% or higher decrease in score from baseline to endpoint | 85% |
|  | Teixeira et al., 2015 | NR | N/R | 29 | HAM-D | Response: 50% or higher decrease in score from baseline to endpoint | 82.80% |
|  | Lowthert et al., 2012 | HAM-D 27.65 | 6.1 | 20 | HAM-D | Response: 50% or higher decrease in score from baseline to endpoint | 50% |
|  | Soeiro-de-Souza et al., 2014 | NR | NR | 25 | HAM-D | Response: 50% or higher decrease in score from baseline to endpoint | 84% |
|  | Machado-Vieira et al., 2014 | HAM-D 22.5 | YMRS 6.1 | 29 | HAM-D | Response: 50% or higher decrease in score from baseline to endpoint | 86.20% |
|  | De Sousa et al., 2015 | HAM-D 22.2 | YMRS 5.6 | 27 | HAM-D | Response: 50% or higher decrease in score from baseline to endpoint | 85% |
|  | De Sousa et al., 2013 | HAM-D 22.5 | YMRS 6.1 | 29 | HAM-D | Response: 50% or higher decrease in score from baseline to endpoint | 86.20% |
|  | Amsterdam et al 2015 | HAM-D 18.8 | YMRS 0.6 | 64 | HAM-D + CGI-S | 50% or more HRSD decrease from baseline to week 12 + final CGI-S score of 3 or less | 34.40% |
|  | Altshuler et al 2017 | Depressive symptomatology-clinician-rated score 35.2 | YMRS 5.6 | 49 | IDS-C (Inventory of Depressive symptomatology - Clinician rated) + CGI | Response: 50% or higher decrease of score of IDS-C, OR a 2 point or higher decrease in CGI from baseline, for at least two consecutive visits spanning at least a 2 week period | 67.40% |
|  | Machado-Vieira et al., 2016 | HAM-D 22.4 | NR | 20 | HAM-D | Response: 50% or higher decrease in score from baseline to endpoint | 85% |
|  |  |  |  | *Total N = 453* |  |  | *Mean = 68.0%* |
| Depression remission | Machado-vieira 2015 | HAM-D 21.7 | YMRS 4.7 | 24 | HAM-D | Remission: Score of 8 or less | 71% |
|  | Machado-Vieira et al., 2016 | 22.6 HAMD | NR | 23 | HAM-D | Remission: Score of 8 or less | 65.20% |
|  | Amsterdam et al., 2008 | 28.6 HAMD | YMRS 1.2 | 40 | HAM-D 17 | Remission: Score of 8 or less | 15.00% |
|  | Soeiro-de-Souza et al., 2014 | NR | NR | 25 | HAM-D | Remission: Score of 8 or less | 64% |
|  | Machado-Vieira et al., 2014 | HAM-D 22.5 | YMRS 6.1 | 29 | HAM-D | Remission: Score of 8 or less | 62% |
|  | Ananth et al., 2020 | HAM-D 27.3 | NR | 19 | HAM-D | <10 HDRS-24 and >=50% reduction in HDRS-24 from pre to post treatment | 26.30% |
|  |  |  |  | *Total N = 160* |  |  | *Mean = 50.55%* |
| Mania response | Keck et al 2001 | 20.0 HAMD mean | 28.3 mean YMRS | 15 | YMRS | Response: 50% or higher decrease in score from baseline to endpoint | 60% |
|  | Bowden et al., 2005 | 6.3 MADRS | 33.3, YMRS | 98 | YMRS | Response: 50% or higher decrease in score from baseline to endpoint | 75.50% |
|  | Young et al., 2017 | N/A | 26.3 YMRS | 112 | YMRS | Response: 50% or higher decrease in score from baseline to endpoint | 78.60% |
|  | Ichim et al., 2000 | n/r | MRS mean 31.6 | 15 | MRS | Response: 50% or higher decrease in score from baseline to endpoint | 60% |
|  | Ichim et al., 2000 | n/r | MRS mean 31.6 | 15 | BPRS | Response: 50% or higher decrease in score from baseline to endpoint | 27% |
|  | Tandon et al., 1981 | HAM-D 19.9 | mean Beigel's Rating scale for mania 14.36 | 50 | Beigels rating scale | Response: 90% or higher decrease in score from baseline to endpoint | 26% |
|  | Tandon et al., 1981 | HAM-D 19.9 | mean Beigel's Rating scale for mania 14.36 | 50 | Beigels rating scale | Response: 70-90% decrease in score from baseline to endpoint | 52% |
|  | Niufan et al., 2008 | 5.0 (MADRS) | 32.4 (YMRS) | 71 | YMRS | Response: 50% or higher decrease in score from baseline to endpoint | 73.20% |
|  | Li et al., 2008 | 4.8 MADRS | 29.8, YMRS | 77 | YMRS | Response: 50% or higher decrease in score from baseline to endpoint | 59.70% |
|  | Mallinger et al 2008 | nr | nr | 45 | BRMS | Response: less than or 7 on RSMS AND less than or 15 on BRMS after minimum 3 weeks of lithium treatment | 58% |
|  |  |  |  | *Total N =548* |  |  | *Mean = 57%* |
| Mania remission | Bowden et al., 2005 | 6.3 MADRS | 33.3, YMRS | 98 | YMRS | Total score of 12 or less at endpoint | 72.40% |
|  | Young et al., 2017 | N/A | 26.3 YMRS | 112 | YMRS | Total score of 9 or less at endpoint | 69.60% |
|  | Niufan et al., 2008 | 5.0 (MADRS) | 32.4 (YMRS) | 71 | YMRS | Total score of 12 or less at endpoint | 70.40% |
|  | Li et al., 2008 | 4.8 MADRS | 29.8, YMRS | 77 | YMRS | Total score of 12 or less at endpoint | 48.10% |
|  | Li et al., 2008 | 4.8 MADRS | 29.8, YMRS | 77 | YMRS | Total score of 8 or less at endpoint | 32.50% |
|  |  |  |  | *Total N = 435* |  |  | *Mean = 58.6%* |
| Mixed scale response | Selek et al 2013 | HAMD 13.52 | YMRS 7.76 | 24 | HAM-D + YMRS | 50% or higher decrease in score from baseline to endpoint | 50% |
|  | Denicoff et al., 1997 | 7.1 HAMD | 3.3 YMRS | 42 | CGI | % with good response to lithium (marked or mod improv) | 33.30% |
|  | Shansis 2016 | 19.20 (HAMD-21) | 10.04 for YMRS, 7.04 for BRMS, 12.06 for CARS-M. | 68 | HAM-D + YMRS | 50% of higher reduction in symptoms | 22.10% |
|  | Shansis 2016 | 19.20 (HAMD-21) | 10.04 for YMRS, 7.04 for BRMS, 12.06 for CARS-M. | 68 | HAM-D + BRMS | 50% of higher reduction in symptoms | 20.60% |
|  | Shansis 2016 | 19.20 (HAMD-21) | 10.04 for YMRS, 7.04 for BRMS, 12.06 for CARS-M. | 68 | HAM-D + CARS-M | 50% of higher reduction in symptoms | 23.50% |
|  | Ichim et al., 2000 | n/r | MRS mean 31.6 | 15 | CGI | Response: score of 1 or 2 | 27% |
|  | Gao 2020 | 4.09, CGI-S depression | 2.69, CGI-S mania | 53 | CGI | <3 after 2 weeks | 5.66% |
|  |  |  |  | *Total N = 338* |  |  | *Mean = 26%* |
| Mixed scale remission | Selek et al 2013 | HAMD 13.52 | YMRS 7.76 | 24 | HAM-D + YMRS | HAM-D score 7 or less + YMRS score 12 or less | 25% |
|  | Strakowski et al, 2016 | HDRS 14 | YMRS 25 | 19 | HAM-D + YMRS | HAM-D + YMRS score 10 or less | 50% |
|  | Machado-Vieira et al., 2017 | HDRS 22.4 | N/A | 20 | HAM-D + YMRS | HAM-D + YMRS score 8 or less | 60% |
|  | Teixeira et al., 2015 | n/r | N/R | 29 | HAM-D + YMRS | HAM-D + YMRS score 8 or less | 62.10% |
|  | De Sousa et al., 2015 | HAM-D 22.2 | YMRS 5.6 | 27 | HAM-D + YMRS | HAM-D + YMRS score 8 or less | 63% |
|  | Nierenberg et al., 2013 | MADRS 22.4 | YMRS 13 | 141 | CGI-BP-S | Score of 2 or less for 2 months | 26.50% |
|  | De Sousa et al., 2013 | 22.5, HAM-D | 6.1, YMRS | 29 | HAM-D + YMRS | HAM-D + YMRS score 8 or less | 62.10% |
|  | Li et al., 2008 | 4.8 MADRS | 29.8, YMRS | 77 | MADRS + YMRS | MADRS score 8 or less + YMRS score 12 or less | 48.10% |
|  | Machado-Vieira et al., 2016 | 22.4 HAMD | nr | 20 | HAM-D + YMRS | HAM-D + YMRS score 8 or less | 60% |
|  |  |  |  | *Total N = 386* |  |  | *Mean = 51%* |
| Negative response / switch or change in medication for response | Kessing et al., 2012 | nr | nr | 1547 | Switch to/add on of another psychotropic drug | During study period | 72% |
|  | Kessing et al., 2011 | nr | n/r | 1555 | Switch to/add on of another psychotropic drug | During study period | 72% |
|  | Gao 2020 | 4.09, CGI-S depression | 2.69, CGI-S mania | 53 | Switched | Intolerant to Lithium, switched to Divalproex (other study arm) | 15.09% |
|  |  |  |  | Total N = 3155 |  |  | Mean = 53.03% |
| Negative response resulting in hospitalisation | Licht et al 2001 | n/r | n/r | 148 | Recurrence hospitalization | % with BD related hospitalisation in study period | 21.60% |
|  | Kessing et al., 2012 | nr | nr | 3518 | Recurrence hospitalization | % with BD related hospitalisation in study period | 57% |
|  | Kessing et al., 2011 | nr | n/r | 3549 | Recurrence hospitalization | % with BD related hospitalisation in study period | 54% |
|  | Burton 2021 | nr | n/r | 24 | Recurrence hospitalization | % with BD related hospitalisation in study period | 14% |
|  |  |  |  | *Total N = 7239* |  |  | *Mean = 36.7%* |
| Negative response / new mood episode/events | Weisler et al., 2011 | 3.3 (MADRS) | 3.7 YMRS | 364 | Any mood event by end of study | At least 1: initiation of medication to treat a mood event; hospitalisation for a mood event, YMRS or MADRS score of 20 or higher at 2 consecutive assessments, or discontinuation from the study if due to mood event | 40% |
|  | Altamura et al., 2008 | nr | n/r | 39 | Major mood episode recurrence | 4 year follow up | 53.80% |
|  | Amsterdam et al 2015 | 18.8 (HAMD) | 0.6, YMRS | 15 | Relapse (major depressive episode), HRSD + CGI-S | HRSD 14 or more + CGI-S score of 4 or more for minimum 14 days | 26.70% |
|  | Bowden et al., 2003 | HAMD-17 6.7 | 22.3, Mania rating scale | 46 | Intervention for any mood episode | New medication, ECT | 39% |
|  | Hayes et al., 2016 | nr | n/r | 1505 | Treatment failure after 2.05 years | Stop medication, add-on of another mood stabilizer, antipsychotic, antidepressant or benzodiazepine | 75% |
|  |  |  |  | *Total N = 1969* |  |  | *Mean = 47%* |
| Other response / no new intervention required | Kessing et al 2014 | nr | n/r | 715 | Response, early intervention (lithium at first contact) | After 5 years: after 6 months of index purchase of lithium, no polypharmacy and no admission to psychiatric ward/hospital | 13.30% |
|  | Kessing et al 2014 | nr | n/r | 410 | Response, early intervention (lithium after single episode diagnosis) | After 5 years: after 6 months of index purchase of lithium, no polypharmacy and no admission to psychiatric ward/hospital | 13.20% |
|  | Kessing et al 2014 | nr | n/r | 3999 | Response, late intervention (lithium at later contact) | After 5 years: after 6 months of index purchase of lithium, no polypharmacy and no admission to psychiatric ward/hospital | 6.30% |
|  | Kessing et al 2014 | nr | n/r | 4304 | Response, late intervention (lithium at BD diagnosis) | After 5 years: after 6 months of index purchase of lithium, no polypharmacy and no admission to psychiatric ward/hospital | 6.70% |
|  | Kessing et al 2014 | nr | n/r | 715 | Response, early intervention (lithium at first contact) | After 10 years: after 6 months of index purchase of lithium, no polypharmacy and no admission to psychiatric ward/hospital | 8.70% |
|  | Kessing et al 2014 | nr | n/r | 410 | Response, early intervention (lithium after single episode diagnosis) | After 10 years: after 6 months of index purchase of lithium, no polypharmacy and no admission to psychiatric ward/hospital | 10.10% |
|  | Kessing et al 2014 | nr | n/r | 3999 | Response, late intervention (lithium at later contact) | After 10 years: after 6 months of index purchase of lithium, no polypharmacy and no admission to psychiatric ward/hospital | 4.00% |
|  | Kessing et al 2014 | nr | n/r | 4304 | Response, late intervention (lithium at BD diagnosis) | After 10 years: after 6 months of index purchase of lithium, no polypharmacy and no admission to psychiatric ward/hospital | 4.20% |
|  | Licht et al 2001 | n/r | n/r | 148 | No rehospitalization | % who were not rehospitalised in 2 years | 30.40% |
|  | Greil et al., 1997 | n/r | NR | 74 | No hospitalisation | During study | 82% |
|  | Vestergaard et al., 1998 | nr | n/r | 91 | No recurrence or readmission to hospital | During 2 years follow up | 34.00% |
|  | Calabrese et al 2003 | 5.6 (HAMD) | 2.0 MRS | 121 | Completed study without intervention | n/a | 17% |
|  | Calabrese et al 2003 | 5.6 (HAMD) | 2.0 MRS | 120 | No intervention, mania | % of those going 1 year without intervention | 86% |
|  | Calabrese et al 2003 | 5.6 (HAMD) | 2.0 MRS | 120 | No intervention, depression | % of those going 1 year without intervention | 46% |
|  |  |  |  | *Total N = 19530* |  |  | *Mean = 26%* |
| Response NOS | Strakowski et al, 2016 | HDRS 14 | YMRS 25 | 19 | Response | How many showed response, not otherwise specified | 58% |
|  | Serretti et al., 2004 | N/R | N/R | NR | Response | Response is if lithium is needed only and they continue to take it for 3 years without a new episode (and continue to show up for the study) | 56.60% |
|  | Breen et al., 2016 | NR | NR | 16 | Response | Reaching the end of the maintenance phase of the study without relapse = lithium responders | 50% |
|  | Maj et al., 1998 | n/r | NR | 402 | Still taking prophylactic lithium | At follow up, 5 years | 61% |
|  | Maj et al., 2002 | nr | n/r | 53 | Still taking prophylactic lithium | At follow up, 5 years | 56.60% |
|  | Maj et al., 1998 | nr | n/r | 402 | hospital time | a 50% reduction in mean annual time spent in the hospital compared to a reference pretreatment period | 85% |
|  | Maj et al., 2002 | nr | n/r | 30 | Reduced time in hospital | a 50% reduction in mean annual time spent in the hospital compared to a reference pre-treatment period | 56.70% |
|  | Bohlken 2020 | nr | n/r | 1098 | No treatment failure | Within 24 months, no discontinuation of treatment or add-on of either mood stabilizer, antipsychotic, antidepressant drug or benzodiazepine | 23.70% |
|  | Bohlken 2020 | nr | n/r | 1098 | No treatment failure | Within 12 months, no discontinuation of treatment or add-on of either mood stabilizer, antipsychotic, antidepressant drug or benzodiazepine | 32.90% |
|  |  |  |  | *Total N = 3118* |  |  | *Mean = 53%* |
| Other remission / no new episodes | Maj et al., 1998 | nr | n/r | 247 | Illness recurrence | No affective episode during treatment period | 23% |
|  | Maj et al., 2002 | nr | n/r | 30 | No new episodes | No new manic or depressive episodes | 23.30% |
|  | Serretti et al 2000 | n/r | nr | 61 | mood episode prevention | % having no episodes during FU | 21.31% |
|  | Serretti et al 2000 | nr | n/r | 61 | mood episode prevention | % having reduced mood ep frequency during FU | 54.10% |
|  | Vestergaard et al., 1998 | nr | n/r | 91 | No recurrence of affective disorder episode | During 2 years follow up | 80.00% |
|  | Hartong et al., 2003 | 5.23, Bech Rafaelsen Melancholia scale | 1.8, Bech Rafaelsen Mania scale | 44 | mood episode prevention | % completing 2 years without episode | 72.70% |
|  | Altamura et al., 2008 | nr | n/r | 39 | Euthymia / remission | 4 year follow up | 46.20% |
|  | Greil et al., 1997 | nr | n/r | 74 | No recurrence of symptoms | During study | 77% |
|  |  |  |  | *Total N = 647* |  |  | *Mean = 50%* |

Supplementary data 4.6: Changes from protocol

| **Element changed** | **Details of change** |
| --- | --- |
| Title | Changed to accurately reflect inclusion of meta-analysis |
| Anticipated completion date | Changed to accurately reflect inclusion of meta-analysis |
| Inclusion of studies with participants under 18 years of age | We included 3 studies which had a minority of participants under the age of 18 in order to provide a more comprehensive picture of literature on the topic. |
| Inclusion of additional study designs | We included more study designs not mentioned in the protocol, such as Open label lithium studies and register studies in order to provide a more comprehensive picture of literature on the topic. |
| Removal of one Risk of Bias criterion | We did not assess if study question in the included studies were clear and appropriate as we found no variation between studies meaning this data was not informative |
| Removal of several data categories | We did not report ethnicity, previous number of bipolar episodes, illness duration prior to treatment or type of monitoring in the included studies, as this data was not available. |
| Use of Cochrane Risk of Bias tool for all included studies | We used the Cochrane Risk of bias tool for RCT for all studies instead of splitting up RCT from NRT in order to give an appropriate risk of bias rating to non-randomised studies when compared to randomised |
| Did not follow the SWIM method | In the interest of brevity and prioritisation of meta-analytic results, we didn’t fully follow the SWIM method, including doing subanalysis of the binary data. |

Supplementary data 5: Included publications for systematic review and meta-analysis

Altamura, A. C., Mundo, E., Dell’Osso, B., Tacchini, G., Buoli, M., & Calabrese, J. R. (2008). Quetiapine and classical mood stabilizers in the long-term treatment of Bipolar Disorder: A 4-year follow-up naturalistic study. *Journal of Affective Disorders*, *110*(1–2), 135–141. https://doi.org/10.1016/j.jad.2008.01.017

Altinay, M., Karne, H., & Anand, A. (2018). Lithium monotherapy associated clinical improvement effects on amygdala-ventromedial prefrontal cortex resting state connectivity in bipolar disorder. *Journal of Affective Disorders*, *225*, 4–12. https://doi.org/10.1016/j.jad.2017.06.047

Altshuler, L. L., Sugar, C. A., McElroy, S. L., Calimlim, B., Gitlin, M., Keck, P. E., Aquino-Elias, A., Martens, B. E., Fischer, E. G., English, T. L., Roach, J., & Suppes, T. (2017). Switch Rates During Acute Treatment for Bipolar II Depression With Lithium, Sertraline, or the Two Combined: A Randomized Double-Blind Comparison. *The American Journal of Psychiatry*, *174*(3), 266–276. https://doi.org/10.1176/appi.ajp.2016.15040558

Amsterdam, J. D., Lorenzo-Luaces, L., Soeller, I., Li, S. Q., Mao, J. J., & DeRubeis, R. J. (2015). Safety and effectiveness of continuation antidepressant versus mood stabilizer monotherapy for relapse-prevention of bipolar II depression: A randomized, double-blind, parallel-group, prospective study. *Journal of Affective Disorders*, *185*, 31–37. https://doi.org/10.1016/j.jad.2015.05.070

Amsterdam, J. D., & Shults, J. (2008). Comparison of short-term venlafaxine versus lithium monotherapy for bipolar II major depressive episode: A randomized open-label study. *Journal of Clinical Psychopharmacology*, *28*(2), 171–181. https://doi.org/10.1097/JCP.0b013e318166c4e6

Amsterdam, J. D., & Shults, J. (2010). Efficacy and safety of long-term fluoxetine versus lithium monotherapy of bipolar II disorder: A randomized, double-blind, placebo-substitution study. *The American Journal of Psychiatry*, *167*(7), 792–800. https://doi.org/10.1176/appi.ajp.2009.09020284

Ananth, M., Bartlett, E. A., DeLorenzo, C., Lin, X., Kunkel, L., Vadhan, N. P., Perlman, G., Godstrey, M., Holzmacher, D., Ogden, R. T., Parsey, R. V., & Huang, C. (2020). Prediction of lithium treatment response in bipolar depression using 5-HTT and 5-HT1A PET. *European Journal of Nuclear Medicine and Molecular Imaging*, *47*(10), 2417–2428. https://doi.org/10.1007/s00259-020-04681-6

Astaneh, A. N., & Rezaei, O. (2012). Adjunctive treatment with gabapentin in bipolar patients during acute mania. *International Journal of Psychiatry in Medicine*, *43*(3), 261–271. https://doi.org/10.2190/PM.43.3.e

Barbuti, M., Colombini, P., Ricciardulli, S., Amadori, S., Gemmellaro, T., De Dominicis, F., Della Rocca, F., Petrucci, A., Schiavi, E., & Perugi, G. (2021). Treatment adherence and tolerability of immediate- and prolonged-release lithium formulations in a sample of bipolar patients: A prospective naturalistic study. *International Clinical Psychopharmacology*, *36*(5), 230–237. https://doi.org/10.1097/YIC.0000000000000373

Bohlken, J., Riedel-Heller, S., Bauer, M., & Kostev, K. (2021). Bipolar Disorder and Outcomes of Monotherapy with Lithium, Valproate, Quetiapine, Olanzapine, Venlafaxine, and Citalopram. *Pharmacopsychiatry*, *54*(3), 126–130. https://doi.org/10.1055/a-1348-1523

Bowden, C. L., Calabrese, J. R., Sachs, G., Yatham, L. N., Asghar, S. A., Hompland, M., Montgomery, P., Earl, N., Smoot, T. M., DeVeaugh-Geiss, J., & Lamictal 606 Study Group. (2003). A placebo-controlled 18-month trial of lamotrigine and lithium maintenance treatment in recently manic or hypomanic patients with bipolar I disorder. *Archives of General Psychiatry*, *60*(4), 392–400. https://doi.org/10.1001/archpsyc.60.4.392

Bowden, C. L., Grunze, H., Mullen, J., Brecher, M., Paulsson, B., Jones, M., Vågerö, M., & Svensson, K. (2005). A randomized, double-blind, placebo-controlled efficacy and safety study of quetiapine or lithium as monotherapy for mania in bipolar disorder. *The Journal of Clinical Psychiatry*, *66*(1), 111–121. https://doi.org/10.4088/jcp.v66n0116

Breen, M. S., White, C. H., Shekhtman, T., Lin, K., Looney, D., Woelk, C. H., & Kelsoe, J. R. (2016). Lithium-responsive genes and gene networks in bipolar disorder patient-derived lymphoblastoid cell lines. *The Pharmacogenomics Journal*, *16*(5), 446–453. https://doi.org/10.1038/tpj.2016.50

Burton, C., Mathys, M., & Gutierrez, E. (2021). Comparison of lithium to second generation antipsychotics for the treatment of bipolar disorder in older veterans. *Psychiatry Research*, *303*, 114063. https://doi.org/10.1016/j.psychres.2021.114063

Calabrese, J. R., Bowden, C. L., Sachs, G., Yatham, L. N., Behnke, K., Mehtonen, O.-P., Montgomery, P., Ascher, J., Paska, W., Earl, N., DeVeaugh-Geiss, J., & Lamictal 605 Study Group. (2003). A placebo-controlled 18-month trial of lamotrigine and lithium maintenance treatment in recently depressed patients with bipolar I disorder. *The Journal of Clinical Psychiatry*, *64*(9), 1013–1024. https://doi.org/10.4088/jcp.v64n0906

Clark, H. M., Berk, M., & Brook, S. (1997). A randomized controlled single blind study of the efficacy of clonazepam and lithium in the treatment of acute mania. *Human Psychopharmacology: Clinical and Experimental*, *12*(4), 325–328. https://doi.org/10.1002/(SICI)1099-1077(199707/08)12:4<325::AID-HUP856>3.0.CO;2-H

Dalkilic, A., Diaz, E., Baker, C. B., Pearsall, H. R., & Woods, S. W. (2000). Effects of divalproex versus lithium on length of hospital stay among patients with bipolar disorder. *Psychiatric Services (Washington, D.C.)*, *51*(9), 1184–1186. https://doi.org/10.1176/appi.ps.51.9.1184

de Sousa, R. T., Zanetti, M. V., Talib, L. L., Serpa, M. H., Chaim, T. M., Carvalho, A. F., Brunoni, A. R., Busatto, G. F., Gattaz, W. F., & Machado-Vieira, R. (2015). Lithium increases platelet serine-9 phosphorylated GSK-3β levels in drug-free bipolar disorder during depressive episodes. *Journal of Psychiatric Research*, *62*, 78–83. https://doi.org/10.1016/j.jpsychires.2015.01.016

de Sousa, R. T., Zarate, C. A., Zanetti, M. V., Costa, A. C., Talib, L. L., Gattaz, W. F., & Machado-Vieira, R. (2014). Oxidative stress in early stage Bipolar Disorder and the association with response to lithium. *Journal of Psychiatric Research*, *50*, 36–41. https://doi.org/10.1016/j.jpsychires.2013.11.011

Denicoff, K. D., Smith-Jackson, E. E., Disney, E. R., Ali, S. O., Leverich, G. S., & Post, R. M. (1997). Comparative prophylactic efficacy of lithium, carbamazepine, and the combination in bipolar disorder. *The Journal of Clinical Psychiatry*, *58*(11), 470–478. https://doi.org/10.4088/jcp.v58n1102

Gao, K., Arnold, J. G., Prihoda, T. J., Quinones, M., Singh, V., Schinagle, M., Conroy, C., D’Arcangelo, N., Bai, Y., Calabrese, J. R., & Bowden, C. L. (2020). Sequential Multiple Assignment Randomized Treatment (SMART) for Bipolar Disorder at Any Phase of Illness and at least Mild Symptom Severity. *Psychopharmacology Bulletin*, *50*(2), 8–25.

Gao, K., Goto, T., Yuan, C., Brownrigg, B., Conroy, C., Chan, P. K., Serrano, M. B., Ganocy, S. J., Fang, F., & Calabrese, J. R. (2018). A Pilot Study of the Effectiveness of Lithium Versus Quetiapine Immediate Release Monotherapy in Patients With Bipolar Spectrum Disorders. *Journal of Clinical Psychopharmacology*, *38*(5), 422–434. https://doi.org/10.1097/JCP.0000000000000927

Greil, W., Ludwig-Mayerhofer, W., Erazo, N., Schöchlin, C., Schmidt, S., Engel, R. R., Czernik, A., Giedke, H., Müller-Oerlinghausen, B., Osterheider, M., Rudolf, G. A., Sauer, H., Tegeler, J., & Wetterling, T. (1997). Lithium versus carbamazepine in the maintenance treatment of bipolar disorders—A randomised study. *Journal of Affective Disorders*, *43*(2), 151–161. https://doi.org/10.1016/s0165-0327(96)01427-9

Hartong, E. G. T. M., Moleman, P., Hoogduin, C. A. L., Broekman, T. G., Nolen, W. A., & LitCar Group. (2003). Prophylactic efficacy of lithium versus carbamazepine in treatment-naive bipolar patients. *The Journal of Clinical Psychiatry*, *64*(2), 144–151. https://doi.org/10.4088/jcp.v64n0206

Hayes, J. F., Marston, L., Walters, K., Geddes, J. R., King, M., & Osborn, D. P. J. (2016). Lithium vs. valproate vs. olanzapine vs. quetiapine as maintenance monotherapy for bipolar disorder: A population-based UK cohort study using electronic health records. *World Psychiatry: Official Journal of the World Psychiatric Association (WPA)*, *15*(1), 53–58. https://doi.org/10.1002/wps.20298

Hollander, E., Pallanti, S., Allen, A., Sood, E., & Baldini Rossi, N. (2005). Does sustained-release lithium reduce impulsive gambling and affective instability versus placebo in pathological gamblers with bipolar spectrum disorders? *The American Journal of Psychiatry*, *162*(1), 137–145. https://doi.org/10.1176/appi.ajp.162.1.137

Ichim, L., Berk, M., & Brook, S. (2000). Lamotrigine compared with lithium in mania: A double-blind randomized controlled trial. *Annals of Clinical Psychiatry: Official Journal of the American Academy of Clinical Psychiatrists*, *12*(1), 5–10. https://doi.org/10.1023/a:1009066725103

Keck, P. E., Strakowski, S. M., Hawkins, J. M., Dunayevich, E., Tugrul, K. C., Bennett, J. A., & McElroy, S. L. (2001). A pilot study of rapid lithium administration in the treatment of acute mania. *Bipolar Disorders*, *3*(2), 68–72. https://doi.org/10.1034/j.1399-5618.2001.030204.x

Kessing, L. V., Hellmund, G., & Andersen, P. K. (2012). An observational nationwide register based cohort study on lamotrigine versus lithium in bipolar disorder. *Journal of Psychopharmacology (Oxford, England)*, *26*(5), 644–652. https://doi.org/10.1177/0269881111414091

Kessing, L. V., Hellmund, G., Geddes, J. R., Goodwin, G. M., & Andersen, P. K. (2011). Valproate v. lithium in the treatment of bipolar disorder in clinical practice: Observational nationwide register-based cohort study. *The British Journal of Psychiatry: The Journal of Mental Science*, *199*(1), 57–63. https://doi.org/10.1192/bjp.bp.110.084822

Kessing, L. V., Vradi, E., & Andersen, P. K. (2014). Starting lithium prophylaxis early v. Late in bipolar disorder. *The British Journal of Psychiatry: The Journal of Mental Science*, *205*(3), 214–220. https://doi.org/10.1192/bjp.bp.113.142802

Li, H., Ma, C., Wang, G., Zhu, X., Peng, M., & Gu, N. (2008). Response and remission rates in Chinese patients with bipolar mania treated for 4 weeks with either quetiapine or lithium: A randomized and double-blind study. *Current Medical Research and Opinion*, *24*(1), 1–10. https://doi.org/10.1185/030079908x253933

Licht, R. W., Vestergaard, P., Rasmussen, N. A., Jepsen, K., Brodersen, A., & Hansen, P. E. (2001). A lithium clinic for bipolar patients: 2-year outcome of the first 148 patients. *Acta Psychiatrica Scandinavica*, *104*(5), 387–390. https://doi.org/10.1034/j.1600-0447.2001.00389.x

Lowthert, L., Leffert, J., Lin, A., Umlauf, S., Maloney, K., Muralidharan, A., Lorberg, B., Mane, S., Zhao, H., Sinha, R., Bhagwagar, Z., & Beech, R. (2012). Increased ratio of anti-apoptotic to pro-apoptotic Bcl2 gene-family members in lithium-responders one month after treatment initiation. *Biology of Mood & Anxiety Disorders*, *2*, 15. https://doi.org/10.1186/2045-5380-2-15

Machado-Vieira, R., Gattaz, W. F., Zanetti, M. V., De Sousa, R. T., Carvalho, A. F., Soeiro-de-Souza, M. G., Leite, C. C., & Otaduy, M. C. (2015). A Longitudinal (6-week) 3T 1H-MRS Study on the Effects of Lithium Treatment on Anterior Cingulate Cortex Metabolites in Bipolar Depression. *European Neuropsychopharmacology*, *25*(12), 2311–2317. https://doi.org/10.1016/j.euroneuro.2015.08.023

Machado-Vieira, R., Otaduy, M. C., Zanetti, M. V., De Sousa, R. T., Dias, V. V., Leite, C. C., Forlenza, O. V., Busatto, G. F., Soares, J. C., & Gattaz, W. F. (2016). A Selective Association between Central and Peripheral Lithium Levels in Remitters in Bipolar Depression: A 3T-(7) Li Magnetic Resonance Spectroscopy Study. *Acta Psychiatrica Scandinavica*, *133*(3), 214–220. https://doi.org/10.1111/acps.12511

Machado-Vieira, R., Zanetti, M., de Sousa, R., Soeiro-de-Souza, M., Moreno, R., Busatto, G., & Gattaz, W. (2014). Lithium efficacy in bipolar depression with flexible dosing: A six-week, open-label, proof-of-concept study. *Experimental and Therapeutic Medicine*, *8*, 1205–1208. https://doi.org/10.3892/etm.2014.1864

Machado-Vieira, R., Zanetti, M. V., Otaduy, M. C., De Sousa, R. T., Soeiro-de-Souza, M. G., Costa, A. C., Carvalho, A. F., Leite, C. C., Busatto, G. F., Zarate, C. A., & Gattaz, W. F. (2017a). Increased Brain Lactate During Depressive Episodes and Reversal Effects by Lithium Monotherapy in Drug-Naive Bipolar Disorder: A 3-T 1H-MRS Study. *Journal of Clinical Psychopharmacology*, *37*(1), 40–45. https://doi.org/10.1097/JCP.0000000000000616

Machado-Vieira, R., Zanetti, M. V., Otaduy, M. C., De Sousa, R. T., Soeiro-de-Souza, M. G., Costa, A. C., Carvalho, A. F., Leite, C. C., Busatto, G. F., Zarate, C. A., & Gattaz, W. F. (2017b). Increased Brain Lactate During Depressive Episodes and Reversal Effects by Lithium Monotherapy in Drug-Naive Bipolar Disorder: A 3-T 1H-MRS Study. *Journal of Clinical Psychopharmacology*, *37*(1), 40–45. https://doi.org/10.1097/JCP.0000000000000616

Maj, M., Pirozzi, R., Bartoli, L., & Magliano, L. (2002). Long-term outcome of lithium prophylaxis in bipolar disorder with mood-incongruent psychotic features: A prospective study. *Journal of Affective Disorders*, *71*(1–3), 195–198. https://doi.org/10.1016/s0165-0327(01)00350-0

Maj, M., Pirozzi, R., Magliano, L., & Bartoli, L. (1998). Long-term outcome of lithium prophylaxis in bipolar disorder: A 5-year prospective study of 402 patients at a lithium clinic. *The American Journal of Psychiatry*, *155*(1), 30–35. https://doi.org/10.1176/ajp.155.1.30

Mallinger, A. G., Thase, M. E., Haskett, R., Buttenfield, J., Luckenbaugh, D. A., Frank, E., Kupfer, D. J., & Manji, H. K. (2008). Verapamil augmentation of lithium treatment improves outcome in mania unresponsive to lithium alone: Preliminary findings and a discussion of therapeutic mechanisms. *Bipolar Disorders*, *10*(8), 856–866. https://doi.org/10.1111/j.1399-5618.2008.00636.x

McNamara, R. K., Jandacek, R., Tso, P., Blom, T. J., Welge, J. A., Strawn, J. R., Adler, C. M., DelBello, M. P., & Strakowski, S. M. (2015). First-Episode Bipolar Disorder is Associated with Erythrocyte Membrane Docosahexaenoic Acid Deficits: Dissociation from Clinical Response to Lithium or Quetiapine. *Psychiatry Research*, *230*(2), 447–453. https://doi.org/10.1016/j.psychres.2015.09.035

Moore, G. J., Bebchuk, J. M., Parrish, J. K., Faulk, M. W., Arfken, C. L., Strahl-Bevacqua, J., & Manji, H. K. (1999). Temporal dissociation between lithium-induced changes in frontal lobe myo-inositol and clinical response in manic-depressive illness. *The American Journal of Psychiatry*, *156*(12), 1902–1908. https://doi.org/10.1176/ajp.156.12.1902

Moore, G. J., Cortese, B. M., Glitz, D. A., Zajac-Benitez, C., Quiroz, J. A., Uhde, T. W., Drevets, W. C., & Manji, H. K. (2009). A longitudinal study of the effects of lithium treatment on prefrontal and subgenual prefrontal gray matter volume in treatment-responsive bipolar disorder patients. *The Journal of Clinical Psychiatry*, *70*(5), 699–705. https://doi.org/10.4088/JCP.07m03745

Nierenberg, A. A., Friedman, E. S., Bowden, C. L., Sylvia, L. G., Thase, M. E., Ketter, T., Ostacher, M. J., Leon, A. C., Reilly-Harrington, N., Iosifescu, D. V., Pencina, M., Severe, J. B., & Calabrese, J. R. (2013). Lithium treatment moderate-dose use study (LiTMUS) for bipolar disorder: A randomized comparative effectiveness trial of optimized personalized treatment with and without lithium. *The American Journal of Psychiatry*, *170*(1), 102–110. https://doi.org/10.1176/appi.ajp.2012.12060751

Niufan, G., Tohen, M., Qiuqing, A., Fude, Y., Pope, E., McElroy, H., Ming, L., Gaohua, W., Xinbao, Z., Huichun, L., & Liang, S. (2008). Olanzapine versus lithium in the acute treatment of bipolar mania: A double-blind, randomized, controlled trial. *Journal of Affective Disorders*, *105*(1–3), 101–108. https://doi.org/10.1016/j.jad.2007.04.020

Pae, C.-U., Drago, A., Kim, J.-J., Mandelli, L., Patkar, A., Ronchi, D., & Serretti, A. (2008). HSP70 variations in the acute treatment with mood stabilizers in patients with bipolar disorder: Results of a preliminary work. *Gene Therapy and Molecular Biology*, *12*, 267–276.

Pal Singh, G. (2008). A double-blind comparative study of clinical efficacy of verapamil versus lithium in acute mania. *International Journal of Psychiatry in Clinical Practice*, *12*(4), 303–308. https://doi.org/10.1080/13651500802209670

Parker, G., Ricciardi, T., Tavella, G., & Hadzi-Pavlovic, D. (2021). A Single-Blind Randomized Comparison of Lithium and Lamotrigine as Maintenance Treatments for Managing Bipolar II Disorder. *Journal of Clinical Psychopharmacology*, *41*(4), 381–388. https://doi.org/10.1097/JCP.0000000000001424

Rucci, P., Frank, E., Kostelnik, B., Fagiolini, A., Mallinger, A. G., Swartz, H. A., Thase, M. E., Siegel, L., Wilson, D., & Kupfer, D. J. (2002). Suicide attempts in patients with bipolar I disorder during acute and maintenance phases of intensive treatment with pharmacotherapy and adjunctive psychotherapy. *The American Journal of Psychiatry*, *159*(7), 1160–1164. https://doi.org/10.1176/appi.ajp.159.7.1160

Segal, J., Berk, M., & Brook, S. (1998). Risperidone compared with both lithium and haloperidol in mania: A double-blind randomized controlled trial. *Clinical Neuropharmacology*, *21*(3), 176–180.

Selek, S., Nicoletti, M., Zunta-Soares, G. B., Hatch, J. P., Nery, F. G., Matsuo, K., Sanches, M., & Soares, J. C. (2013). A longitudinal study of fronto-limbic brain structures in patients with bipolar I disorder during lithium treatment. *Journal of Affective Disorders*, *150*(2), 629–633. https://doi.org/10.1016/j.jad.2013.04.020

Serretti, A., Lattuada, E., Franchini, L., & Smeraldi, E. (2000). Melancholic features and response to lithium prophylaxis in mood disorders. *Depression and Anxiety*, *11*(2), 73–79. https://doi.org/10.1002/(sici)1520-6394(2000)11:2<73::aid-da4>3.0.co;2-a

Serretti, A., Malitas, P. N., Mandelli, L., Lorenzi, C., Ploia, C., Alevizos, B., Nikolaou, C., Boufidou, F., Christodoulou, G. N., & Smeraldi, E. (2004). Further evidence for a possible association between serotonin transporter gene and lithium prophylaxis in mood disorders. *The Pharmacogenomics Journal*, *4*(4), 267–273. https://doi.org/10.1038/sj.tpj.6500252

Shafti, S. S. (2018). Aripiprazole Versus Lithium in Management of Acute Mania: A Randomized Clinical Trial. *East Asian Archives of Psychiatry: Official Journal of the Hong Kong College of Psychiatrists = Dong Ya Jing Shen Ke Xue Zhi: Xianggang Jing Shen Ke Yi Xue Yuan Qi Kan*, *28*(3), 80–84.

Shansis, F. M., Reche, M., & Capp, E. (2016). Evaluating response to mood stabilizers in patients with mixed depression: A study of agreement between three different mania rating scales and a depression rating scale. *Journal of Affective Disorders*, *197*, 1–7. https://doi.org/10.1016/j.jad.2016.02.064

Shoja Shafti, S., & Kaviani, H. (2018). Extended-release carbamazepine versus lithium in management of acute mania in male inpatients with bipolar I disorder. *Psychiatry and Clinical Psychopharmacology*, *28*(4), 371–377. https://doi.org/10.1080/24750573.2018.1449181

Silverstone, P. H., Bell, E. C., Willson, M. C., Dave, S., & Wilman, A. H. (2005). Lithium alters brain activation in bipolar disorder in a task- and state-dependent manner: An fMRI study. *Annals of General Psychiatry*, *4*, 14. https://doi.org/10.1186/1744-859X-4-14

Simhandl, C., Denk, E., & Thau, K. (1993). The comparative efficacy of carbamazepine low and high serum level and lithium carbonate in the prophylaxis of affective disorders. *Journal of Affective Disorders*, *28*(4), 221–231. https://doi.org/10.1016/0165-0327(93)90057-q

Singh, L. K., Nizamie, S. H., Akhtar, S., & Praharaj, S. K. (2011). Improving tolerability of lithium with a once-daily dosing schedule. *American Journal of Therapeutics*, *18*(4), 288–291. https://doi.org/10.1097/MJT.0b013e3181d070c3

Soeiro-de-Souza, M. G., Gold, P. W., Brunoni, A. R., de Sousa, R. T., Zanetti, M. V., Carvalho, A. F., Gattaz, W. F., Machado-Vieira, R., & Teixeira, A. L. (2014). Lithium decreases plasma adiponectin levels in bipolar depression. *Neuroscience Letters*, *564*, 111–114. https://doi.org/10.1016/j.neulet.2014.02.005

Spielberg, J., Matyi, M., Karne, H., & Anand, A. (2018). Lithium Monotherapy Associated Longitudinal Effects on Resting State Brain Networks in Clinical Treatment of Bipolar Disorder. *Bipolar Disorders*, *21*. https://doi.org/10.1111/bdi.12718

Strakowski, S. M., Fleck, D. E., Welge, J., Eliassen, J. C., Norris, M., Durling, M., Komoroski, R. A., Chu, W.-J., Weber, W., Dudley, J. A., Blom, T. J., Stover, A., Klein, C., Strawn, J. R., DelBello, M. P., Lee, J.-H., & Adler, C. M. (2016). FMRI brain activation changes following treatment of a first bipolar manic episode. *Bipolar Disorders*, *18*(6), 490–501. https://doi.org/10.1111/bdi.12426

Suppes, T., Marangell, L. B., Bernstein, I. H., Kelly, D. I., Fischer, E. G., Zboyan, H. A., Snow, D. E., Martinez, M., Al Jurdi, R., Shivakumar, G., Sureddi, S., & Gonzalez, R. (2008). A single blind comparison of lithium and lamotrigine for the treatment of bipolar II depression. *Journal of Affective Disorders*, *111*(2–3), 334–343. https://doi.org/10.1016/j.jad.2008.02.004

Tandon, A. K., Asare, R., & Saxena, V. C. (1981). Lithium treatment in affective disorders. *Indian Journal of Psychiatry*, *23*(1), 58–61.

Teixeira, A. L., de Sousa, R. T., Zanetti, M. V., Brunoni, A. R., Busatto, G. F., Zarate, C. A., Gattaz, W. F., & Machado-Vieira, R. (2015). Increased plasma levels of soluble TNF receptors 1 and 2 in bipolar depression and impact of lithium treatment. *Human Psychopharmacology*, *30*(1), 52–56. https://doi.org/10.1002/hup.2450

Tondo, L., Baldessarini, R. J., Floris, G., & Rudas, N. (1997). Effectiveness of restarting lithium treatment after its discontinuation in bipolar I and bipolar II disorders. *The American Journal of Psychiatry*, *154*(4), 548–550. https://doi.org/10.1176/ajp.154.4.548

Vestergaard, P., Licht, R. W., Brodersen, A., Rasmussen, N. A., Christensen, H., Arngrim, T., Grønvall, B., Kristensen, E., & Poulstrup, I. (1998). Outcome of lithium prophylaxis: A prospective follow-up of affective disorder patients assigned to high and low serum lithium levels. *Acta Psychiatrica Scandinavica*, *98*(4), 310–315. https://doi.org/10.1111/j.1600-0447.1998.tb10089.x

Weisler, R. H., Nolen, W. A., Neijber, A., Hellqvist, A., Paulsson, B., & Trial 144 Study Investigators. (2011). Continuation of quetiapine versus switching to placebo or lithium for maintenance treatment of bipolar I disorder (Trial 144: A randomized controlled study). *The Journal of Clinical Psychiatry*, *72*(11), 1452–1464. https://doi.org/10.4088/JCP.11m06878

Young, R. C., Mulsant, B. H., Sajatovic, M., Gildengers, A. G., Gyulai, L., Al Jurdi, R. K., Beyer, J., Evans, J., Banerjee, S., Greenberg, R., Marino, P., Kunik, M. E., Chen, P., Barrett, M., Schulberg, H. C., Bruce, M. L., Reynolds, C. F., Alexopoulos, G. S., & GERI-BD Study Group. (2017). GERI-BD: A Randomized Double-Blind Controlled Trial of Lithium and Divalproex in the Treatment of Mania in Older Patients With Bipolar Disorder. *The American Journal of Psychiatry*, *174*(11), 1086–1093. https://doi.org/10.1176/appi.ajp.2017.15050657
